# Quantitative Characterization of Organosilane Monolayers by Oxidative Dissociation of Monolayer Molecules

**DOI:** 10.1021/acs.analchem.4c06937

**Published:** 2025-02-22

**Authors:** Naeem Iqbal, Amy Wolstenholme-Hogg, James R. Gompels, Victor Chechik

**Affiliations:** Department of Chemistry, University of York, York YO10 5DD, U.K.

## Abstract

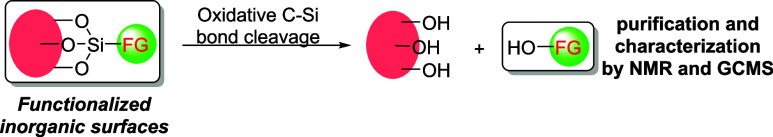

Self-assembled organosilane monolayers on silica surfaces find many applications; however, their structural characterization is challenging. We found that organic molecules in these monolayers can be dissociated from the surface by cleaving C–Si bonds under mild conditions of Fleming-Tamao oxidation. Once removed from the surface, the monolayer molecules could be isolated, purified, and analyzed in solution using conventional analytical techniques including NMR and GC-MS. This method enables efficient cleavage of different organic molecules attached to silica supports (e.g., in mixed monolayers) and is tolerant to a wide range of functional groups. Organic monolayers can be dissociated from a range of silica substrates, including silica nanoparticles, silica gel, flat glass slides, and related inorganic oxides, such as alumina or titania.

## Introduction

Silica surfaces or supports, including porous or nonporous (nano)particles, can be modified with a monolayer of specific organic functionalities via a stable Si–C covalent linkage.^1,2^ This is most commonly achieved by treating the surfaces with functional trichloro- or trialkoxysilanes.^3−5^ Due to the propensity of these compounds to self-condense and polymerize, such modification is sometimes difficult to control and reproduce. There are however many reports in the literature providing protocols for making good quality, well-characterized monolayers from trichloro-/trialkoxysilanes.^6,7^ Self-polymerization can be avoided by using monochloro-/monoalkoxydimethylsilanes.^8,9^ However, this method is rarely used, and the vast majority of modified silicas continue to be prepared from trichloro-/trialkoxysilanes.

**Scheme 1:**
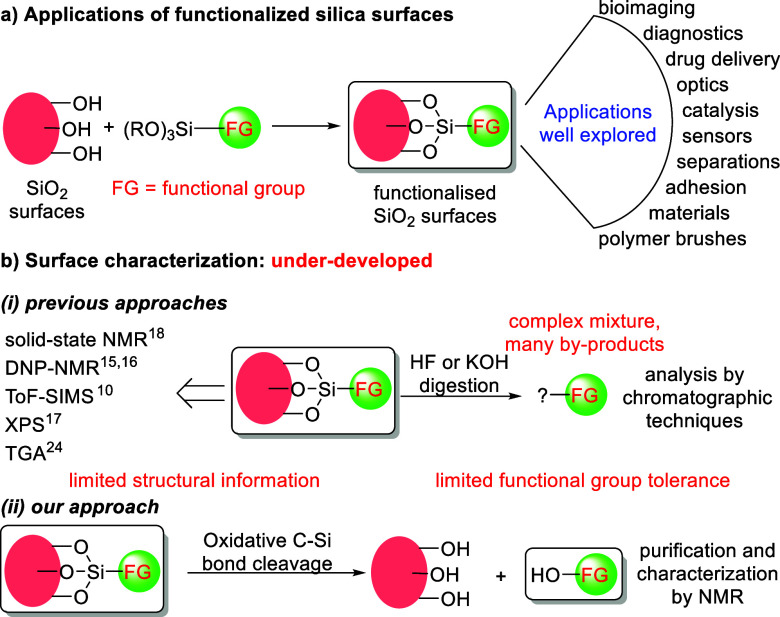
Applications and Characterization of Functionalized Silica Surfaces

Such modified silicas have attracted significant attention due to their wide-ranging applications in many diverse fields such as bioimaging, drug delivery, optics, catalysis, polymer brushes (Scheme 1a).^10−14^ Structural and quantitative characterization of organic monolayers on silica surfaces is crucial for the development of existing functional materials and the rational design of new ones. While the design complexity of these hybrid systems continues to grow, methods for characterizing the organic compounds attached to their surfaces have received less attention (Scheme 1b).

Some monolayer-coated substrates can be characterized in situ, e.g., by solid-state NMR techniques, including dynamic nuclear polarization solid-state NMR.^15,16^ These methods can confirm the presence of organic functional groups and provide evidence of certain linkages such as C–Si bonds. However, NMR approaches suffer from low sensitivity (e.g., they can only be applied to small nanoparticles where an organic monolayer constitutes a significant proportion of the overall sample). Thermogravimetric analysis makes it possible to quantify the amount of organic coating on the nanoparticles. Coupling this method with FT-IR or MS can provide some structural information about the adsorbed molecules. Other techniques such as time-of-flight secondary ion mass spectrometry (ToF-SIMS), X-ray photoelectron spectroscopy (XPS) also do not provide detailed quantitative information about the exact nature of the organic moiety attached to the solid support (Scheme 1b(i)).^10,17−19^

Alternatively, organic monolayers can be characterized by dissociating the molecules from the solid substrates. The monolayer molecules released into solution can then be studied by using conventional analytical techniques. For instance, organic Au-thiol monolayers can be readily dissociated from the Au surface as disulfides by treatment with an oxidizing agent in the presence of a suitable ligand (e.g., O_2_ + CN^–^ or I_2_ + I_3_^–^).^20,21^ Dissociation of organosilane monolayers from SiO_2_ surfaces, however, is more challenging. Conventional methods include bond cleavage by digestion of hybrid materials in HF, or under basic conditions (KOH), followed by chromatographic separation.^22,23^ These approaches often result in complex mixtures, which complicate quantitative analysis. In addition, their scope is reduced due to the use of toxic HF and corrosive KOH. For instance, quantitative NMR coupled with KOH/NaOD cleavage has recently been employed for quantitative and qualitative characterization of surface-bonded molecules.^24−26^ However, the use of KOH/NaOD limited its application to a small range of base-stable substrates (Scheme 1b(i)).

Despite these shortcomings, chemical cleavage of monolayers followed by spectroscopic or chromatographic characterization remains an attractive approach, as it can give more detailed and accurate structural and quantitative information about the monolayer composition than in situ methods. In order to extend the applicability of these methods to a wider range of materials, mild conditions for the cleavage of surface-bound organosilanes with good functional group tolerance need to be developed. We hypothesized that the C–Si bond in organosilane monolayers can be cleaved under the Fleming-Tamao oxidation conditions. This would remove the surface-attached molecules to give an alcohol as a dissociated product which can be isolated in pure form and characterized.^27^ A similar approach has previously been employed to cleave short alkylsilanes bound to high-performance liquid chromatographic (HPLC) silica stationary phases and for the cleavage of immobilized molecules from glass substrates.^28,29^ However, literature reports provide limited examples of the substrates (mainly containing amide groups); they operate at elevated temperatures and give low yields of dissociated products, which makes quantitative analysis challenging. Here, we report a general organosilane cleavage protocol based on the Fleming-Tamao oxidation reaction. The protocol can be used for quantification of monolayer composition (e.g., for mixed monolayers), it operates at room temperature under mild conditions, and is applicable to a broad range of substrates (Scheme 1b(ii)).

## Experimental Part

### General monolayer dissociation procedure

Functionalized particles (100 mg) were added to a solution of KHCO_3_ (8.0 equiv) and Bu_4_NF (8.0 equiv) in THF (HPLC grade, 10 mL) and stirred for 3 h under nitrogen (equivalents are relative to the expected amount of organic chains in the monolayer). 30% aq. H_2_O_2_ (12.0 equiv) was added, and the reaction mixture was allowed to stir overnight at room temperature under inert atmosphere. In order to quench basic byproducts and remove fluoride ions, Dowex 50WX8 200–400 (1.6 g) and CaCO_3_ (400 mg) were added, and the resulting solution was stirred for another 30 min. The reaction mixture was filtered through a plug of Celite and washed with DCM. Addition of Dowex and CaCO_3_ was omitted for the acid sensitive functional groups such as ketone **2g**, and ester **2j**. The solution was concentrated on a rotary evaporator. For quantification, an internal standard was added prior to ^1^H NMR analysis. For isolation, the crude product was purified by flash column chromatography using hexane/ethyl acetate (MeOH/DCM for **2s**) as the eluent.

For dissociation of monolayers from the Petri dish and GC analysis, a solution of KHCO_3_ (14 mg), and Bu_4_NF (128 μL, 1 M in THF) in THF (6 mL) was stirred for 15 min. The solution was transferred to a functionalized Petri dish using a syringe and the Petri dish was sealed for 3 h. Thirty % aq. H_2_O_2_ (15 μL) was added and sealed overnight. The solution was then concentrated on a rotary evaporator. The concentrated solution was dissolved in the minimum amount of diethyl ether and filtered through a silica plug with diethyl ether/ethyl acetate mixture (3:1). The filtrate was collected and evaporated to dryness. *N*,*O*-Bis(trimethylsilyl)trifluoroacetamide (BSTFA, 100 μL) was added followed by pyridine (20 μL) and the mixture was heated at 100 °C for 30 min.

## Results and Discussion

### Optimisation of monolayer cleavage

**Scheme 2:**
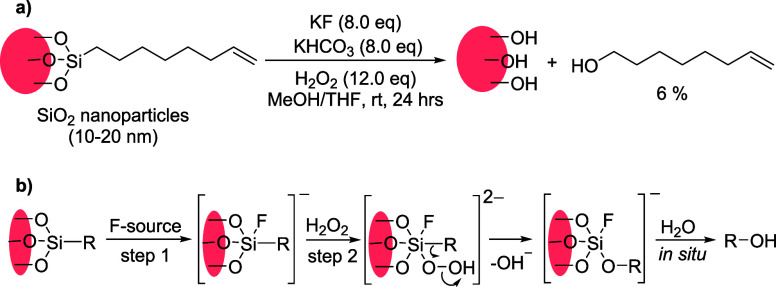
Initial Conditions of C–Si Bond Cleavage and Mechanism of Fleming-Tamao Oxidation

Nonporous silica nanoparticles (10–20 nm) with BET surface area of 219 m^2^/g were selected for the development of monolayer dissociation conditions (Section S2, S4). These relatively small particles have a large surface area and the quantity of organic components released after dissociation is more than sufficient for analysis by conventional techniques such as NMR. The particles were coated with a trimethoxy(7-octen-1-yl)silane monolayer at ca. 0.95 mmol/g (ca. 2.5 molec nm^–2^) coverage (Sections S5–S6).^11^ We started our investigation by applying the established Fleming-Tamao oxidation conditions to the monolayer dissociation (Scheme 2a, equivalents are relative to the expected number of organic chains in the monolayer). This reaction proceeds in two steps. Initially, a pentacoordinate intermediate is formed by the attack of a fluoride ion on the Si center. An attack by an oxidizing agent gives a hexacoordinate structure which undergoes a rearrangement to yield a silanol which under aqueous conditions forms the final product as alcohol (Scheme 2b).^27,30^ Functionalized silica nanoparticles were treated with KF in the presence of KHCO_3_, followed by the addition of H_2_O_2_ as an oxidant and stirring for 24 h in a MeOH/THF solvent system at room temperature, yielding the dissociated alkene in 6% yield (Scheme 2a; the yields were calculated by determining the organic content in each sample by thermogravimetric (TGA) and/or elemental analyses, SI sections 4–5). We note toxicity of KF; however fluoride salts are nonvolatile which significantly simplifies their handling.

We then attempted to optimize the two steps of the Fleming-Tamao reaction individually (Table 1) by (i) stirring the nanoparticles with KF and KHCO_3_ for 24 h, and (ii) subsequently adding H_2_O_2_ as an oxidant and stirring for another 24 h in the MeOH/THF solvent system, resulting in a 20% yield of the dissociated alkene (Table 1, entry 1). Increasing the temperature of the first step, considered to be the rate-limiting step, led to a slightly lower yield of the dissociated product (entry 2). Substituting THF with MeCN and DCM did not yield improved results (entries 3–4).

**Table 1:**
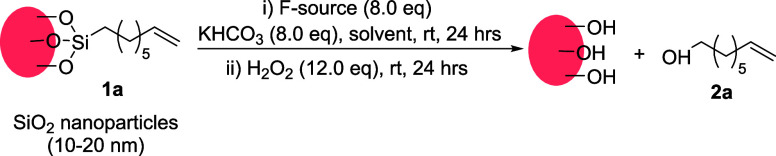
Optimisation of C–Si Bond Cleavage^a^

No	F-Source (8.0 equiv)	Solvent	Variations	Yield (%)^b^
1	KF	MeOH/THF	-	20
2	KF	MeOH/THF	Step 1 at 80 °C	15
3	KF	MeOH/MeCN	-	19
4	KF	MeOH/DCM	-	5
5	NH_4_F	MeOH/THF	-	0
6	TBAF	MeOH/THF	-	32
7	TBAF	THF	-	83
8	TBAF	THF	Step 1 for 3 h	91
9	TBAF	THF	All reagents added at the same time	75
10	TBAF (4.0 equiv)	THF	KHCO_3_ (4.0 equiv), H_2_O_2_ (6.0 equiv)	67
11	TBAF (2.0 equiv)	THF	KHCO_3_ (2.0 equiv), H_2_O_2_ (3.0 equiv)	53
12	TBAF	THF	H_2_O_2_ (15.0 equiv)	92
13	-	THF	-	trace
14	TBAF	THF	no H_2_O_2_	0
15	TBAF	THF	no KHCO_3_	51

a Reactions were conducted by using 100 mg of functionalized silica nanoparticles (0.095 mmol of alkene) in 10 mL of solvent.

b ^1^H NMR yields (internal standard: dimethyl terephthalate).

Since the reaction occurs at the solid–liquid interface, switching the fluoride source from KF to TBAF improved the yield due to the higher solubility of TBAF in organic solvents (entries 5–6). We hypothesized that protic solvents would stabilize F^–^ by hydrogen bonding, thus reducing its nucleophilicity and hindering the dissociation process. Indeed, employing THF as the sole solvent instead of a MeOH/THF mixture significantly increased the yield of the desired product (entry 7). Shortening the duration of the first step from 24 to 3 h improved the efficiency of C–Si bond cleavage (entry 8). Simultaneously adding the fluoride source and the oxidant resulted in decreased reaction efficiency (entry 9). Reducing the amounts of TBAF and H_2_O_2_ also led to a decreased yield of the alcohol (entries 10–11). However, increasing the quantity of the oxidizing agent did not affect the product yield (entry 12). Control experiments confirmed that both the fluoride source and the oxidizing agent are necessary to cleave the C–Si bond (entries 13–14). The yield was also compromised when dissociation was performed in the absence of base (entry 15). The overall optimized conditions (entry 8) are mild (e.g., reaction proceeds at ambient temperature, a significant improvement compared to previous reports which necessitate elevated temperatures).^24,25^ Utilization of TBAF as a fluoride source, as opposed to KF, also improves the safety profile of the reaction.

### Functional group tolerance

With optimized conditions in hand, we proceeded to explore their tolerance to a range of functional groups (Table 2). Silica nanoparticles (10–20 nm) were functionalized with various trialkoxysilanes (Sections S3, S7) and subjected to the optimized dissociation conditions. Saturated alkane/alkene chains were dissociated in high yields (**2a**–**b**). However, the benzyl monolayer yielded the corresponding alcohol (**2c**) in low yield, possibly due to partial oxidation of the dissociated product to an aldehyde or an acid. Aryl group-containing monolayers were dissociated in excellent yields regardless of their size (**2d**–**e**). Organic moieties comprising different functional groups such as ether (**2f**), ketone (**2g**), and nitrile (**2h**) were also successfully dissociated.

**Table 2:**
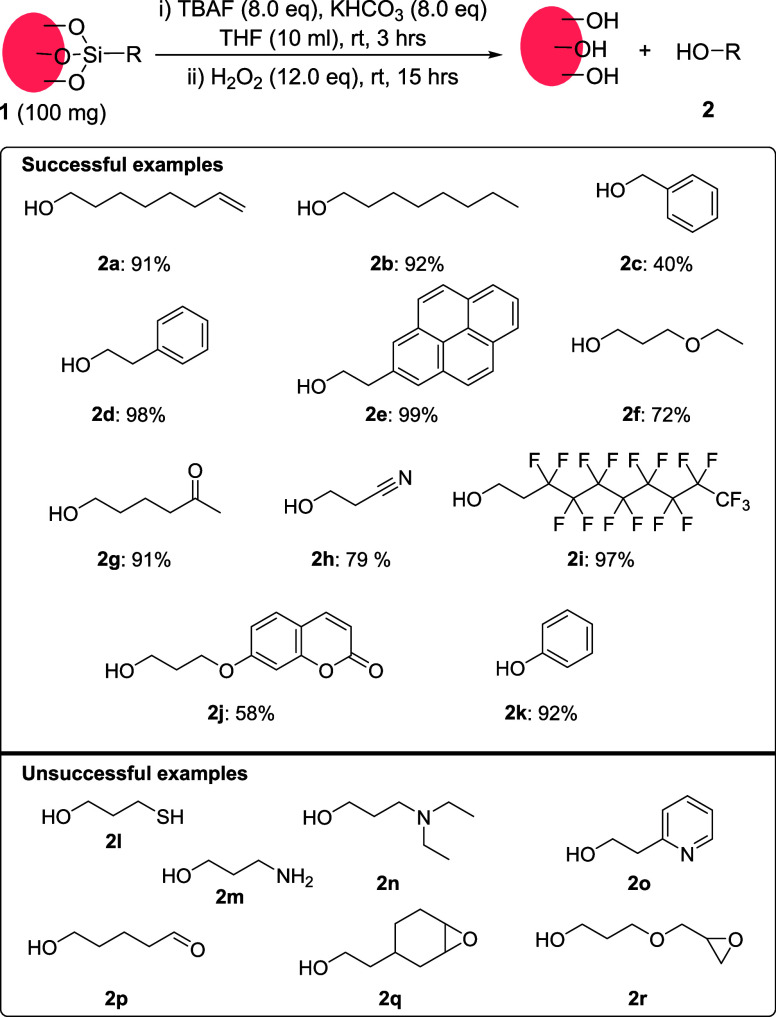
Substrate Scope for C–Si Bond Cleavage^a^

a ^1^H NMR yields (internal standard: dimethyl terephthalate); the yields are based on the organic content as determined by TGA and/or elemental analyses, Sections S5–S6.

Perfluoroalkylated monolayer was also dissociated to give the corresponding alcohol (**2i**) in an excellent yield.^31,32^ Similarly, a photoactive coumarin derivative was dissociated in a moderate yield (**2j**),^33^ while a phenyl monolayer was cleaved to yield phenol (**2k**) as the dissociated product in nearly quantitative yield. We note that the reaction mixtures of dissociated monolayers have relatively small amounts of byproducts, which makes it possible to isolate the dissociated molecules in pure form. For instance, pure alcohols (**2a**–**b**, **2i, 2k**, **2s**) were isolated from the reaction mixtures by column chromatography and fully characterized (Section S7). This has not been achieved in previous reports.

Although the optimized protocol tolerates a wide range of functional groups, several oxidation-prone functionalities were unsuitable for dissociation (Table 2). Thiols became oxidized under these conditions (**2l**). Some nitrogen-containing compounds such as primary amine (**2m**), tert-amine (**2n**), and pyridine (**2o**) were also incompatible with the dissociation conditions, likely undergoing *N*-oxidation in the presence of hydrogen peroxide. Alternative methods described in the introduction section (e.g., dissolution of silica in KOH/NaOH followed by NMR analysis) could be used for monolayer characterization in these cases.^22^ In order to validate the complementarity of this approach to our method, we digested nanoparticles **1a** in NaOH. NMR analysis showed an 84% yield of dissociated alkene (Section S8). We note that unlike our method, NaOH digestion makes it possible to characterize the functional groups only, there is no possibility of separation and full characterization of the dissociated product. We believe this is a significant disadvantage, particularly for complex systems and mixed monolayers. Additionally, aldehyde (**2p**) and epoxides (**2q**–**r**) did not withstand the dissociation conditions probably due to the oxidation of the aldehyde and ring opening of the epoxide in the presence of H_2_O_2_ under basic conditions.

**Scheme 3:**
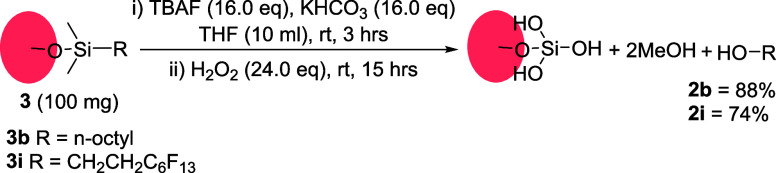
Dissociation of Monolayer Formed by Monoalkoxysilanes

In order to test the applicability of our approach to the monolayers prepared from monoalkoxysilanes,^8,9^ we functionalized silica nanoparticles with two model dimethylmethoxysilanes (**3b**, **3i**). Initial results gave a low yield of dissociated products (ca. 50%). We noted, however, that dimethylalkoxysilane derivatives need to undergo three consecutive Fleming-Tamao oxidations involving two methyl groups as well as the target group. Therefore, they require higher quantities of oxidizing reagents. Doubling the amount of the reagents in the dissociation mixture gave improved yields of the dissociated molecules (Scheme 3).

**Scheme 4:**
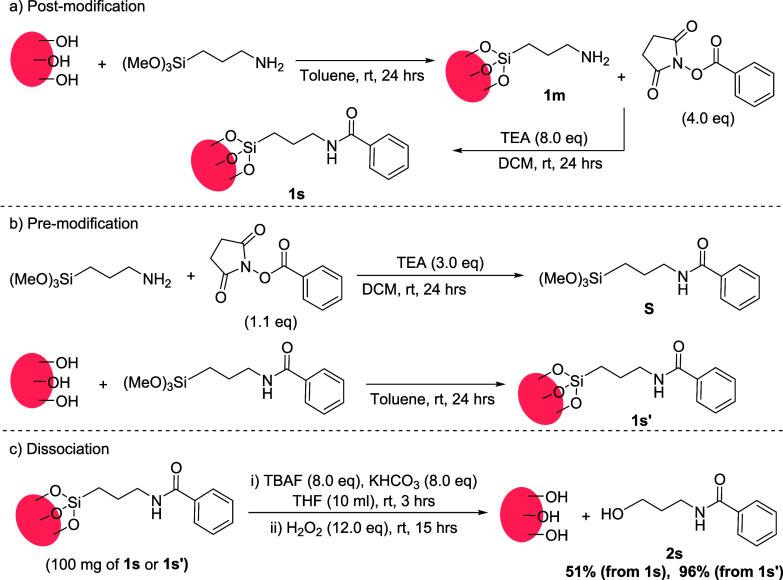
Amide Linkage on Silica Surfaces and Its Dissociation

Aminopropyltrimethoxysilane (APTMS) is commonly used for modification of silica surfaces with amino groups.^34−36^ These groups then serve as anchoring points for attaching desired functionalities, typically through amide bond formation, e.g., in biomedical applications. However, quantitative characterization of the amide linkages prepared by this postmodification approach is challenging. To test the suitability of our method for probing the efficiency of postmodification, we dissociated both postmodified monolayers and those prepared directly with presynthesized amide molecules. Silica nanoparticles were functionalized with APTMS and surface amines **1m** were converted into benzamides **1s** by coupling with *N*-(benzoyloxy)succinimide (Scheme 4a). In parallel, silica nanoparticles were directly functionalized with preprepared benzoylated APTMS (**S**) to give nanoparticles **1s′** (Scheme 4b). Both post- (**1s**) and premodified (**1s’**) silica nanoparticles were then subjected to dissociation conditions.

We found that the postmodification approach gave a moderate yield of surface amide, with only 51% of the amide detected after dissociation. In contrast, the nanoparticles coated with the presynthesized amide (**1s′**) underwent complete dissociation, with 95% of the amide detected after dissociation. This observation suggests that while postmodification is convenient for covalently attaching desired molecules to amine-terminated surfaces, the yield of attachment is not always high. We did not attempt to improve the yield in the postfunctionalization approach by optimizing the reaction conditions.

**Scheme 5:**
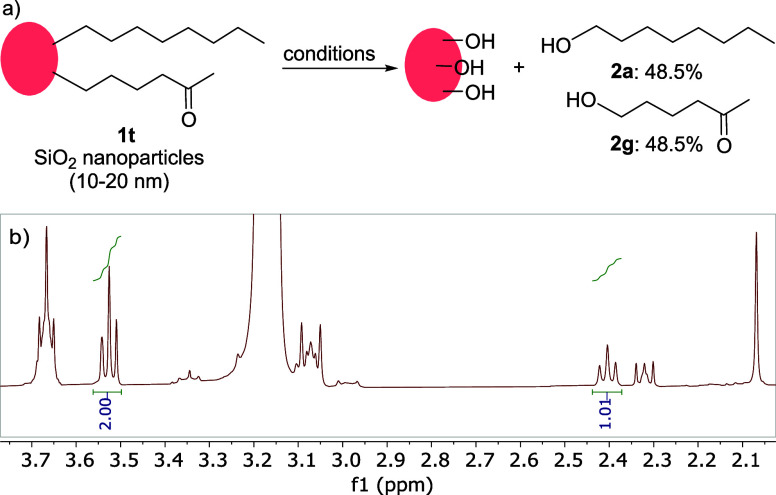
Dissociation of Mixed Monolayer from Different Silica Surfaces and Crude ^1^H NMR Spectrum of the Dissociated Ketone/Alkane (50:50)

Some applications require the presence of several different functional groups on the same surface. In these cases, silica surfaces are functionalized with a mixture of different trichloro- or trialkoxysilanes, yielding a mixed monolayer.^37,38^ Mixed monolayers can also form following an incomplete chemical reaction of functionalized nanoparticles, for instance, in a biological system. However, quantitative analysis of the composition of the mixed monolayers is difficult. We therefore explored the suitability of our approach to the characterization of mixed monolayers. Silica nanoparticles were functionalized with alkane and ketone trialkoxysilanes in a 1:1 ratio (Scheme 5, **1t**) and subjected to the dissociation conditions.

A facile cleavage of mixed monolayers was observed with an overall yield of 97% (Scheme 5a). The ketone:alkane ratio can be calculated from the crude ^1^H NMR spectra (Scheme 5b). Although the spectra show strong contamination peaks (mostly tetrabutylammonium and its decomposition products), the peaks for the dissociated products are clearly visible in the spectra. For example, dissociation mixtures for pure alkane and pure ketone both showed a triplet at ca. 3.50 ppm, corresponding to the α-CH_2_ of the alcohol. The α-CH_2_ of the carbonyl group in the ketone at 2.40 ppm was then used to calculate the ratio of the two monolayer components as 1:1. We note that the composition of mixed monolayers of compounds which do not form strong intermolecular bonds, often reflects the composition of the deposition solution.^39^

### Substrate scope

**Scheme 6:**
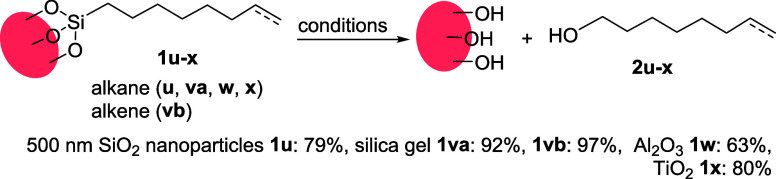
Dissociation from Different Solid Surfaces

Larger functionalized silica nanoparticles (500 nm) with smaller surface area (BET surface area: 12 m^2^/g, Section S2) and low loading of organic content (0.095 mmol/g, sections S5–S6) can also be characterized by the dissociation approach (Scheme 6, **1u**), showcasing the sensitivity of the method (i.e., a monolayer-coated sample with ca. 0.6 m^2^ area, such as 100 mg of 500 nm nanoparticles, is sufficient for NMR analysis). Monolayers on high surface area porous silica (e.g., BET surface area: 333 m^2^/g, 0.75 mmol/g organic content loading for alkane **1va** and alkene **1vb**) were also dissociated in excellent yields. These results suggest that our method can be applied to the characterization of silica particles with different sizes and morphologies.

Furthermore, the method was tested to characterize functionalized metal oxide supports such as titania^40−42^ (BET surface area: 2.5 m^2^/g, 0.056 mmol/g) and alumina^43,44^ (BET surface area: 13 m^2^/g, 0.53 mmol/g) surfaces. The dissociated products were detected in both cases, but we note a lower yield for alumina (Scheme 6).

**Scheme 7:**
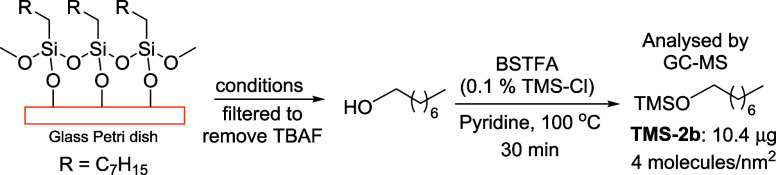
Dissociation of Octanol from a Glass Petri Dish

One of the major applications of surface functionalization is in sensors,^45,46^ which often include functional monolayers on planar substrates with exceptionally small surface area. The qualitative and quantitative analysis of such monolayers is very challenging. In order to test the feasibility of detecting dissociated monolayer on a planar support, we used a 9 cm diameter Petri dish functionalized with octyltrimethoxysilane. The amount of organic material in this monolayer is insufficient for NMR analysis; hence, GC-MS was used for detection after monolayer dissociation. In order to reduce the tailing of the octanol peak, the dissociated alcohol was silylated using BSTFA (*N*,*O*-bis(trimethylsilyl)trifluoroacetamide, Scheme 7). Control experiments showed that dissociated alcohol can be quantitatively detected using our analytical procedures (Section S9). Quantification of the Petri dish dissociation product (Section S10) showed 10.4 μg of dissociated protected octanol which (assuming quantitative yield of dissociation) corresponds to surface coverage of 4 molecules/nm^2^, close to the literature reports.^47^ This method can thus be applied to qualitative and quantitative analyses of the monolayers on planar silica substrates. We estimate that our method with GC-MS analysis can be used to quantify a monolayer in a sample with ca. 60 cm^2^ area (e.g., 1 mg of 500 nm nanoparticles with 12 m^2^/g surface area or a 6 × 10 cm planar substrate).

## Conclusion

In conclusion, we have developed a general method for the qualitative and quantitative analysis of organic monolayers on silica supports through the oxidative cleavage of C–Si bonds. The developed method makes it possible to isolate and purify dissociated organic molecules. The optimized dissociation conditions showed good functional group tolerance, they can be used for analysis of mixed monolayers and are compatible with a range of silica substrates (e.g., with different size and morphology) including planar substrates. The method can be used to quantitatively assess the yields of chemical reactions in monolayers, as demonstrated by using an amide coupling example. The main limitation is that the method cannot be applied to monolayers that are prone to oxidation (e.g., many nitrogen-containing compounds) or are hydrolytically unstable (e.g., esters, epoxides). Despite this limitation, we believe our approach will be a valuable addition to the monolayer characterization toolset applicable to a range of different applications.

## Supporting Information Available

Additional experimental details, materials and methods, characterization data (PDF)
