# Supplementary material for: Quantitative Characterization of Organosilane Monolayers by Oxidative Dissociation of Monolayer Molecules
Source: Anal Chem. 2025 Feb 22;97(8):4661–7. doi: 10.1021/acs.analchem.4c06937 (PMC11883733; doi:10.1021/acs.analchem.4c06937)
Supplement: Supplementary file 1 — ac4c06937_si_001.pdf [file ac4c06937_si_001.pdf]

Supporting Information

Quantitative Characterisation of Organosilane Monolayers by Oxidative Dissociation of Monolayer Molecules

Naeem Iqbal, Amy Wolstenholme-Hogg, James R. Gompels, and Victor Chechik*

Department of Chemistry, University of York, Heslington, York YO10 5DD, United Kingdom
E-mail: victor.chechik@york.ac.uk

Table of Content

	Page No.
**S1. Reagents and Methods**	S2
**S2. Nanoparticle characterisation**	S3
**S3. Experimental Details**	S4
**S4. BET Surface Area Analysis**	S10
**S5. Elemental Analysis**	S11
**S6. Thermogravimetric Analysis**	S13
**S7. Analytic Data for Synthesized Compounds**	S18
**S8. NaOH digestion of nanoparticles 1a**	S21
**S9. Quantitative detection of octanol by ^{1}H NMR and GC-MS**	S22
**S10. GC-MS data of monolayer 1b digestion from Petri dish**	S23
**S11. References**	S24
**S12. NMR Spectra (^{1}H NMR, ^{13}C NMR and ^{19}F NMR)**	S25

The raw data for this work can be found at https://doi.org/10.5281/zenodo.14887519

S1. Reagents and Methods

S1.1 General Reagent Information

All commercially available reagents were purchased from Sigma-Aldrich, Alfa Aesar, Fluorochem or TCI and were used without further purification. Silica nanoparticles (10-20 nm) TiO_2, and Al_2O_3 were purchased form Sigma Aldrich. 500 nm silica nanoparticles were purchased form SkySpring Nanomaterials, Inc. Flash column chromatography was performed using Supelco silica gel 60 (220-440 mesh).

S1.2 General Analytical Information

The specific surface areas (SSA) of the samples were estimated from Brunauer–Emmett–Teller (BET) theory by using nitrogen adsorption–desorption isotherm data obtained at − 196 °C (77 K) on a constant-volume adsorption apparatus with The Micromeritics® TriStar II Plus. The samples were degassed at 200 °C for 12 hours (3 hours for Al_2O_3) before BET analysis. The functionalized silica nanoparticles (10-20 nm and 500 nm), and other solid surfaces (silica gel, TiO_2 and Al_2O_3) were characterized by TGA and elemental analysis. Thermogravimetric analysis (TGA) was performed on the PL Thermal Sciences STA 625 (Simultaneous Thermal Analyser) at the University of York. TGA was done under a flow of nitrogen with a temperature gradient of 10 ^{o}C per minute from room temperature to 625 ^{o}C. Elemental analysis was performed by Elementar vario MICRO cube at University of Sheffield. The synthesized triethoxysilane molecules (**E**, **F**, **G**, **J**, **S**) and dissociated alcohols, **2a**, **2b**, **2i**, **2k** and **2s** were characterized by ^{1}H, and ^{13}C{^{1}H}, and ^{19}F NMR spectroscopy. NMR spectra were recorded on a Varian 400 MHz instrument (400 MHz for ^{1}H NMR, 101 MHz for ^{13}C{^{1}H} NMR, 376 MHz for ^{19}F NMR and 79 MHz for ^{29}Si NMR). Copies of ^{1}H NMR, and ^{13}C{^{1}H} NMR spectra can be found in this Supporting Information. ^{1}H NMR experiments are reported in units, parts per million (ppm), and were measured relative to residual chloroform (7.26 ppm) in the deuterated solvent. ^{13}C NMR spectra are reported in ppm relative to deuterochloroform (77.23 ppm), and all were obtained with ^{1}H decoupling. Coupling constants were reported in Hz. GC-MS was performed using a Waters GCT Premier Time of Flight mass spectrometer coupled to an Agilent 7890A GC system. The column (Phenomenex ZB-5MSplus) dimensions were 30.0 m x 250 μm x 0.25 μm (length x internal diameter x thickness) and the carrier gas was helium. GC method: the initial temperature was 60°C and this was ramped to 260 ^{o}C for 25 ^{o}C/minutes, hold time 2 minutes, then ramped at 50 ^{o}C/minutes to 320 ^{o}C. Total time 15 minutes.

S2. Nanoparticle characterisation

Samples	Sg_{BET} (m^2 g^{-1})	Vg_{BJH} (cm^3 g^{-1})	Dv (nm)
SiNPs (10-20 nm)	219.1823	1.048040	25.3828
SiNPs (500 nm)	12.1482	0.044714	18.8862
Silica gel	333.0738	0.565963	6.2780
Al_2O_3	31.3282	0.252391	6.6843
TiO_2	2.5146	0.006382	10.9676

S3. Experimental Details

S3.1 General Procedures:

S3.1.1 Surface functionalisation of silica nanoparticles (1a-1s', 1u, 3b, and 3i).[S1]

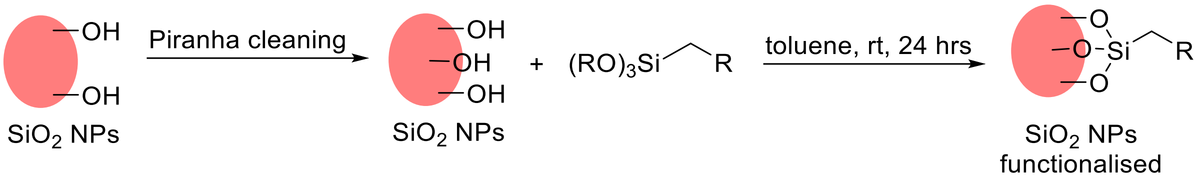

Step 1: The silica nanoparticles (10-20 nm, 500nm) were cleaned for 1 hour in piranha solution (1:3 of 30% H_2O_2 and concentrated H_2SO_4), rinsed three times with Millipore water and centrifuged 3 times, then rinsed with methanol and centrifuged 3 times methanol before being dried under reduced pressure at 40 °C. The substrates were then used immediately for monolayer preparation.

Step 2: The dried particles (1 g) were added to toluene (25 ml) before adding respective trialkoxysilane* (3.0 equivalent to the per mole of surface hydroxyl groups) and stirred at room temperature overnight. The particles were then rinsed with methanol and centrifuged 3 times and dried on a rotary evaporator to get the functionalised nanoparticles.

*Methoxy(dimethyl)octylsilane and Chloro(dimethyl)(3,3,4,4,5,5,6,6,7,7,8,8,8-tridecafluorooctyl)silane were used as precursor instead of trialkoxysilane for the synthesis of **3b** and **3i** respectively.

S3.1.2 Procedure for surface functionalisation of silica nanoparticles with mixed monolayers (1t).

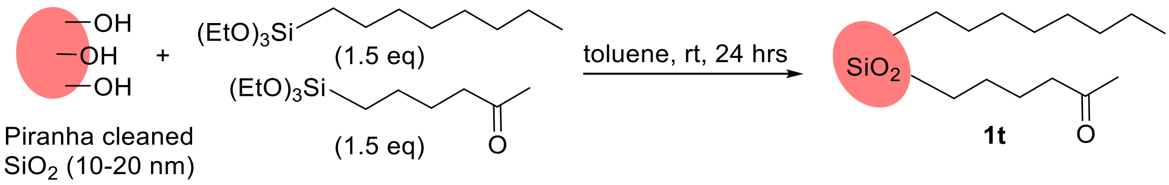

1t

A equimolar solution of n-octyltriethoxysilane (1.5 eq.) and 6-(triethoxysilyl)hexan-2-one (1.5 eq.) was added to the suspension of dried, piranha-cleaned silica nanoparticles (1 g) in toluene (25 ml) under nitrogen. The mixture was stirred at room temperature overnight. The particles were rinsed with methanol and centrifuged 3 times to remove the unreacted trimethoxysilane and dried on a rotary evaporator to get the desired mixed monolayer functionalised nanoparticles.

S3.1.3 Procedure for surface functionalisation of silica gel (1v).

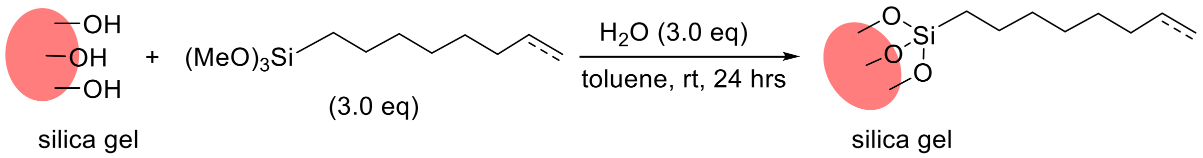

The suspension of silica gel (1 g) in toluene (25 ml) and water (3.0 eq.) was stirred vigorously for 3 hours before adding triethoxy(oct-7-en-1-yl)silane (3.0 eq.) and stirring at room temperature overnight. The particles were then rinsed with methanol and centrifuged 3 times to remove the unreacted trimethoxysilane and dried on a rotary evaporator to get the functionalised silica gel.

S3.1.4 Procedure for surface functionalisation of alumina (1w) and titanium oxide (1x).[S2]

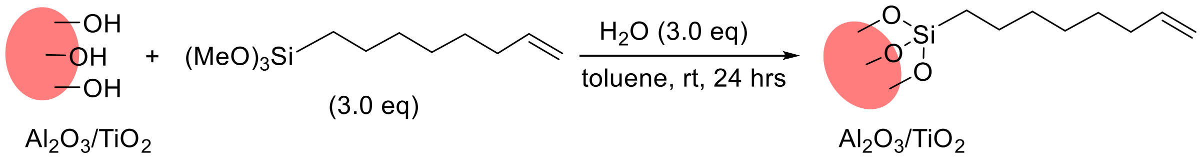

The suspension of particles (1 g) in toluene (25 ml) and triethoxy(oct-7-en-1-yl)silane (3.0 eq.) was refluxed for 16 hours under nitrogen. The particles were then rinsed with methanol and centrifuged 3 times to remove the unreacted trimethoxysilane and dried on a rotary evaporator to get the desired product.

S3.1.5 Amidation of amino terminated silica nanoparticles (1s).[S3]

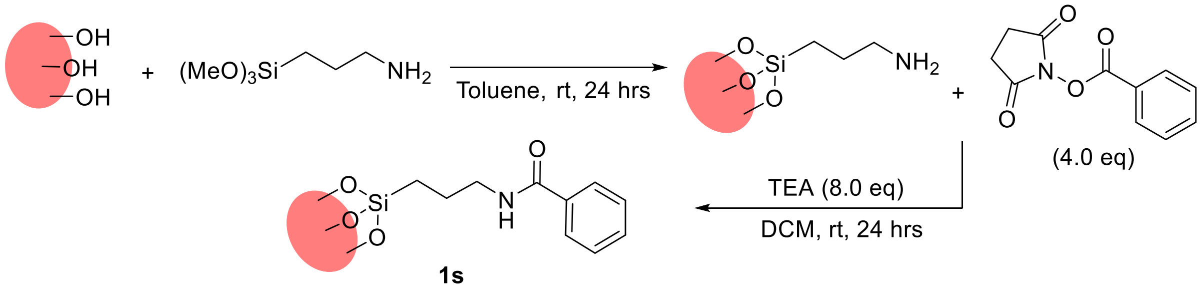

1s

Step 1: The silica nanoparticles (10-20 nm) were cleaned for 1 hour in piranha solution (1:3 of 30% H_2O_2 and concentrated H_2SO_4), rinsed three times with Millipore water and centrifuged 3 times, then rinsed with methanol and centrifuged 3 times methanol before being dried under reduced pressure at 40 ^oC. The substrates were then used immediately for monolayer preparation.

Step 2: The particles (1 g) were then added to toluene (25 ml) before adding aminopropyltrimethoxysilane (3.0 eq.) and stirring at room temperature overnight. The particles were then rinsed with methanol and centrifuged 3 times and dried on a rotary evaporator.

Step 3: Amino terminated silica nanoparticles were dispersed in dry DCM followed by the addition of triethylamine. A solution of 2,5-dioxopyrrolidin-1-yl benzoate (prepared by known

procedure)[S4] was added slowly at 0 °C. After complete addition the mixture was stirred at room temperature overnight. The particles were then rinsed with dichloromethane and centrifuged 3 times to remove the unreacted components and dried on a rotary evaporator.

S3.1.6 Procedure for surface functionalisation of glass Petri dish.

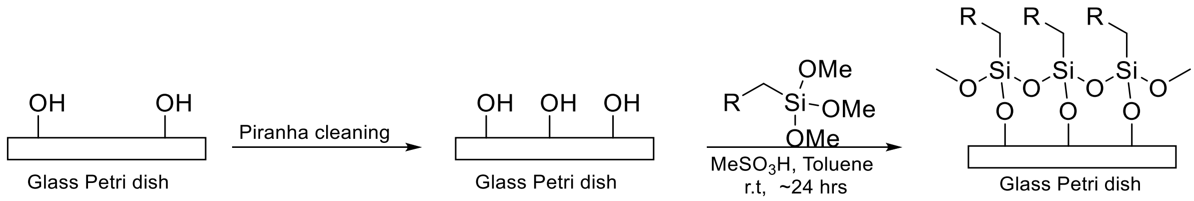

The Petri dish was cleaned for 1 hour in piranha solution (1:3 of 30% H_2O_2 and concentrated H_2SO_4), rinsed several times with deionised water and methanol and then dried with a stream of nitrogen. The substrates were then used immediately for monolayer preparation or placed in deionised water to avoid contamination.

Freshly cleaned substrates were placed in a solution of silane precursor (8 mM), $MeSO_3H$ (0.05mM) in dry toluene (15 ml) and left sealed overnight at room temperature. The substrates were then rinsed with methanol several times and dried using a stream of nitrogen. Functionalisation was confirmed by change in contact angle measurement.

S3.1.7 General Procedure for C-Si bond cleavage.

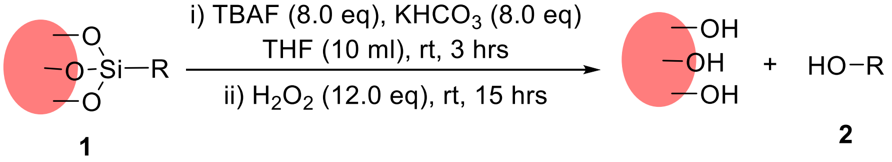

General monolayer dissociation procedure. Functionalised particles (100 mg) are added to a solution of THF (HPLC grade, 10 ml), $KHCO_3$ (8.0 eq.) and Bu_4NF (8.0 eq.) and stirred for 3 hours under nitrogen (equivalents are relative to the expected amount of organic content in the monolayer). H_2O_2 (12.0 eq) was added and reaction mixture was allowed to stir overnight at room temperature under inert atmosphere. In order to quench basic by-products and remove fluoride ions, Dowex 50WX8 200-400 (1.6 g) and $CaCO_3$ (400 mg) were added and resulting solution was stirred for another 30 minutes. The reaction mixture was filtered through a plug of celite and washed with DCM. Addition of Dowex and $CaCO_3$ was omitted for the acid sensitive functional groups such as ketone (**2g**), and esters (**2j**). The particles were then washed with THF 3 times and separated by centrifugation. The solution was concentrated on a rotary evaporator. For quantification, internal standard was added prior to ^{1}H NMR analysis. For isolation, the crude product was purified by flash column chromatography using hexane/ethyl acetate (MeOH/DCM for **2s**) as the eluent.

For dissociation of monolayers from the Petri dish and GC analysis, a solution of THF (6 ml), $KHCO_3$ (14 mg), and Bu_4NF (128 µl, 1M) was stirred for 15 minutes. The solution was transferred to a functionalised Petri dish using a syringe and the Petri dish was sealed for 3 hours. H_2O_2 (15 µl, 30% in water) was added and sealed overnight. The solution was then concentrated on a rotary evaporator. The concentrated solution was dissolved in the minimum amount of diethylether and filtered through a silica plug with diethyl ether/ethyl acetate mixture (3/1). The filtrate was collected and evaporated to dryness. A solution of *N*,*O*-Bis(trimethylsilyl)trifluoroacetamide (BSTFA, 100 µl) was added followed by pyridine (20 µl) and the mixture was heated at 100 °C for 30 minutes.

S3.2 Synthesis of non-commercial trialkoxysilane substrates:

S3.2.1 Synthesis of triethoxy(2-(pyren-1-yl)ethyl)silane (E).[S5]

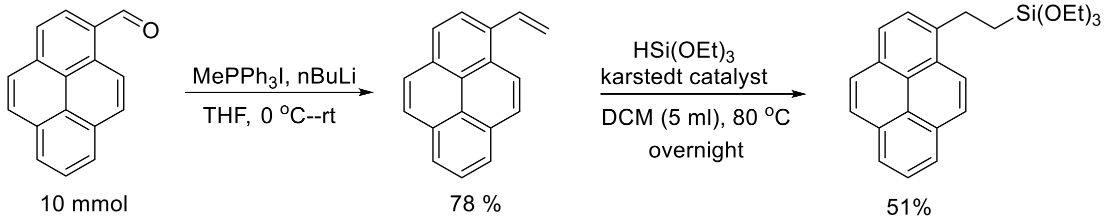

In an oven-dried round-bottom flask, methyltriphenylphosphonium bromide (1.1 eq.) was suspended in dry THF (40 mL) and cooled down to 0 °C. n-BuLi (2.5 M in hexane, 1.1 eq.) was added dropwise to the solution. The reaction mixture was allowed to warm up to room temperature and was stirred for 10 min. 1-Pyrenecarboxaldehyde (10 µmol, 1.0 eq.) dissolved in dry THF (10 mL) was added dropwise. The resulting yellow suspension was stirred overnight (15 hours). After filtration of the reaction mixture, water (50 mL) was added and extracted with ether (3 x 40 mL). The combined organic phase was dried over $MgSO_4$, filtered and concentrated in vacuum. After purification by column chromatography on silica (hexane/ethyl acetate) 1-vinylpyrene (78%) was obtained as a yellow solid.

In an oven dried pressure vessel, 1-vinylpyrene (7.0 mmoles, 1.0 eq.) was suspended in dry DCM (5 mL, just enough to dissolve 1-vinylpyrene) and triethoxysilane (1.0 eq.) was added to the solution under nitrogen. Reaction mixture was purged with nitrogen for 5 minutes. Karstedt catalyst (0.1 mol%) was added under positive pressure of nitrogen. Pressure vessel was sealed and reaction mixture was allowed to stir at 80 °C for 15 hours. The reaction mixture was allowed to coll down to room temperature. The crude product was purified by column chromatography on deactivated silica* using hexane/ethyl acetate eluent to get triethoxy(2-(pyren-1-yl)ethyl)silane (1.6g, 51%) as a yellow viscous liquid.

S3.2.2 Synthesis of triethoxy(3-ethoxypropyl)silane (F)[S6]

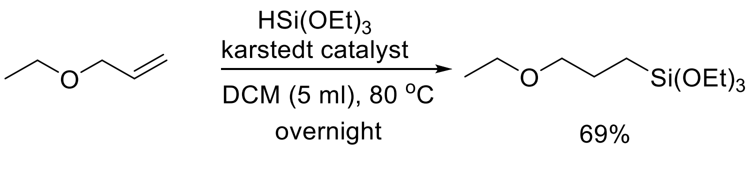

To an oven dried pressure vessel, allyl ether (10.0 mmoles, 1.0 eq.) triethoxysilane (1.0 eq.) were added under nitrogen. Reaction mixture was purged with nitrogen for 5 minutes. Karstedt catalyst (0.1 mol%) was added under positive pressure of nitrogen. Pressure vessel was sealed and reaction mixture was allowed to stir at 80 °C for 15 hours. The reaction mixture was allowed to cool down to room temperature. The crude product was purified by column chromatography on deactivated silica* using hexane/ethyl acetate eluent to get triethoxy(3-ethoxypropyl)silane (1.8g, 58%) as a colorless liquid.

S3.2.3 Synthesis of 6-(triethoxysilyl)hexan-2-one (G)

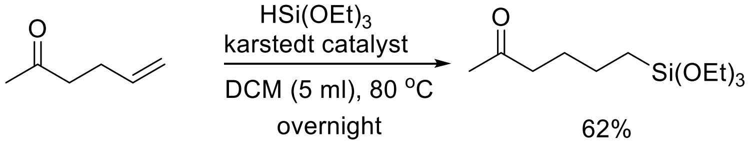

To an oven-dried pressure vessel, hex-5-en-2-one (9.8 mmoles, 1.0 eq.) and triethoxysilane (1.0 eq.) were added under nitrogen. The reaction mixture was purged with nitrogen for 5 minutes. Karstedt catalyst (0.1 mol%) was added under positive pressure of nitrogen. The pressure vessel was sealed and the reaction mixture was allowed to stir at 80 °C for 15 hours. The reaction mixture was allowed to cool down to room temperature. The crude product was purified by column chromatography on deactivated silica* using hexane/ethyl acetate eluent to get 6-(triethoxysilyl)hexan-2-one (1.6g, 62%) as a colorless liquid.

S3.2.4 Synthesis of 7-(3-(triethoxysilyl)propoxy)-2H-chromen-2-one (J).

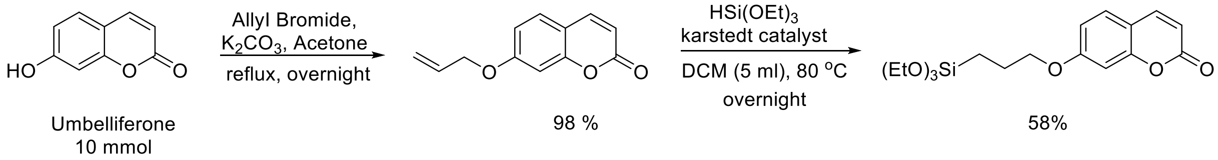

A solution of umbelliferone (10 mmol), allyl bromide (1.2 eq.) and K_2CO_3 (1.2 eq.) in acetone (100 mL) in and oven dried round-bottom flask was heated under reflux for 15 h. Filtration, and evaporation, gave umbelliferyl allyl ether in 98% yield as a colorless solid. The product was used for the next step without further purification.

In an oven dried pressure vessel, umbelliferyl allyl ether (9.8 mmoles, 1.0 eq.) was suspended in dry DCM (5 mL, just enough to dissolve umbelliferyl allyl ether) and triethoxysilane (1.0 eq.) was added to the solution under nitrogen. Reaction mixture was purged with nitrogen for 5 minutes. Karstedt catalyst (0.1 mol%) was added under positive pressure of nitrogen. Pressure vessel was sealed and reaction mixture was allowed to stir at 80 °C for 15 hours. The reaction mixture was allowed to cool down to room temperature. The crude product was purified by column chromatography on deactivated silica* using hexane/ethyl acetate eluent to get 7-(3-(triethoxysilyl)propoxy)-2H-chromen-2-one (2.15g, 58%) as a yellow viscous liquid.

S3.2.5 Synthesis of *N*-(3-(triethoxysilyl)propyl)benzamide (S).

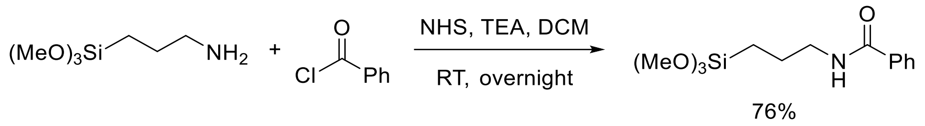

In a 100 mL round-bottom flask, benzoyl chloride (1.1 eq.) was added to 50 mL of dry dichloromethane (DCM) under dry inert conditions. Triethylamine (2.2 eq.) was then added, followed by *N*-hydroxysuccinimide (1.1 eq.), while maintaining the reaction temperature at 0°C under a nitrogen atmosphere. The mixture was allowed to stir at room temperature for 2 hours, then filtered into another oven-dried 100 mL round-bottom flask. Subsequently, a slow addition of aminopropyltrimethoxysilane (5.0 mmols, 1.0 eq.) was made to the filtered mixture, and the reaction was stirred at room temperature for 20 hours. The reaction mixture was filtered again, and the volatiles were evaporated. The resulting crude product was purified using flash column chromatography with a 5% methanol in DCM eluent over deactivated silica gel yielding *N*-(3-(triethoxysilyl)propyl)benzamide as a pale yellow oil (1.24g, 76%).

***Deactivated silica gel**: silica gel was washed with 5% tetraethoxysilane in hexane and excess tetraethoxysilane was removed by using 20% ethylacetate in hexane before crude product loading.

S4. BET surface Area Analysis

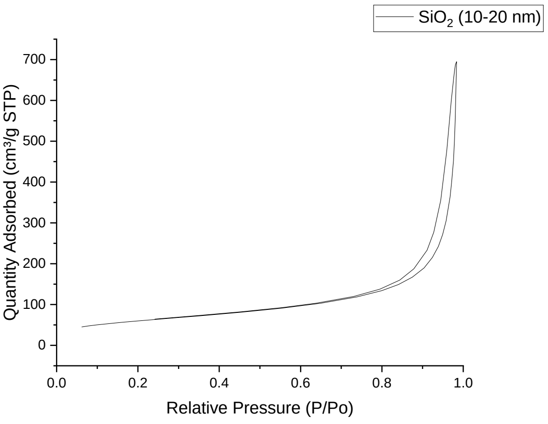

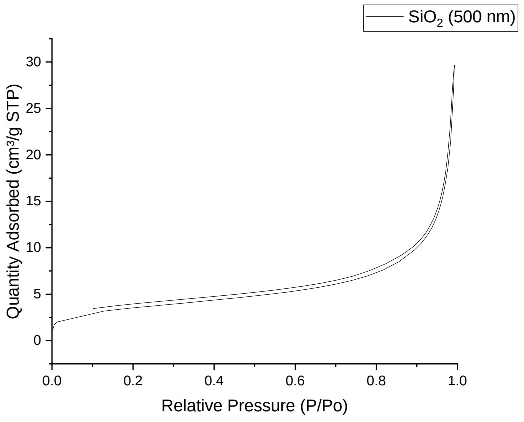

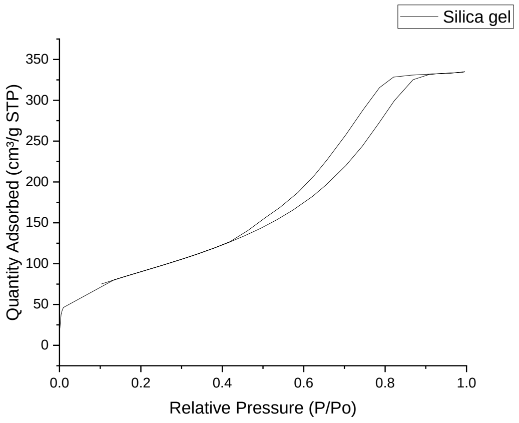

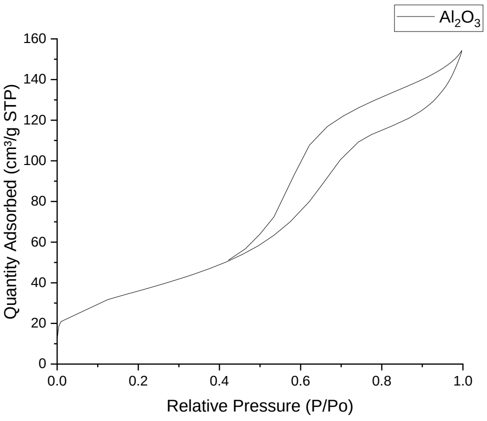

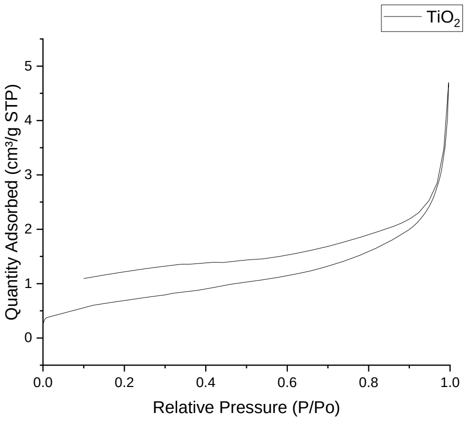

S5. Elemental Analysis

Table S2. Elemental analysis of functionalized particles

**Sample Name**	N (%)	C (%)	H(%)	S(%)	C/H ratio	C/N ratio	Organic content, mmol/g	Organic content, (TGA) mmol/g
**1a**	0.01	9.13	1.44		6.3233		0.95	0.95
**1b**	0.02	9.14	1.636	0	5.58		0.952	0.95
**1c**[a]	0.02	8.65	1.2165	0	10.52		1.038	0.71
**1d**[a]	0.02	9.36	1.081	0	8.6599		0.975	0.63
**1e**[a]	0.02	17.72	1.087	0.157	16.293		0.819	0.57
**1f**	0.01	6.34	1.216	0	5.2105		1.056	1.13
**1g**	0.01	10.44	1.645	0.044	6.35		1.45	1.27
**1h**	1.8	4.93	0.566	0	8.710	2.7409	1.35	1.18
**1i**	0.06	18.02	0.78	0.0	23.06		1.52	1.56
**1j**	0.01	26.89	2.138	0.143	12.58		1.86	1.71
**1k**[a]	0.02	7.56	0.707	0.019	10.6976		1.05	0.626
**1l**	0	5.26	1.04	4.12	5.0587	1.28 (C/S)	1.46	1.19
**1m**	2.99	8.04	2.123	1.381	3.7861	2.6921	2.23	2.46
**1n**	0.9	6.02	1.326	0	4.543	6.7301	0.717	0.65
**1o**[a]	1.39	8.48	1.01	0	8.39	5.4903	1.037	0.84
**1p**	0.01	11.41	1.872	0	6.096		1.90	1.98
**1q**	0.12	12.62	2.13	0	5.92		1.27	1.24
**1r**	0.01	20.6	3.352	0.984	6.146		2.86	2.83
**1s`**	1.7	14.41	1.6	0.06	9.0	8.47	1.10	1.028
**1t**	0.02	5.5	0.901	0	6.1017		0.655	0.57
**1u**[b]	0.01	0.48	0.372	0	1.3		0.05	0.095
**1va**	0.07	6.69	1.79	0	3.74		0.77	0.75
**1vb**	0.006	5.27	1.408	0.035	3.74		0.61	0.75
**1w**	0	5.13	0.73	0	7.03		0.53	0.77
**1x**[b]	0	0.56	0	0	0		0.056	0.09

**3b**	0.02	3.02	0.73	0.13	4.14		0.28	0.25
**3i**[a]	0.57	2.67	0.33	0	8.09		0.257	0.19

[a]Samples containing aromatic groups tend not to burn completely during thermogravimetric analysis. CHN analysis was used to quantify the surface bonded molecules and hence for the yield calculation. [b]Samples with very low loading of organic contents fall in the error range of the elemental analysis instrument, hence TGA data was used to quantify the surface bonded molecules and subsequently for yield calculation.

S6. Thermogravimetric Analysis.

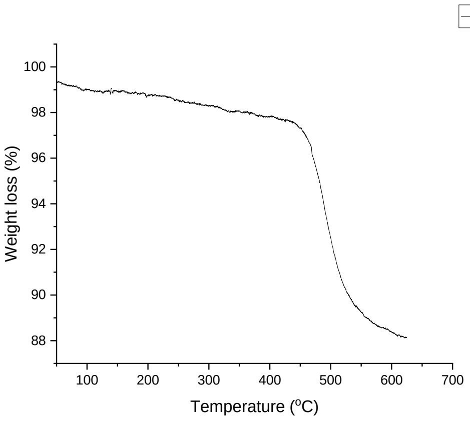

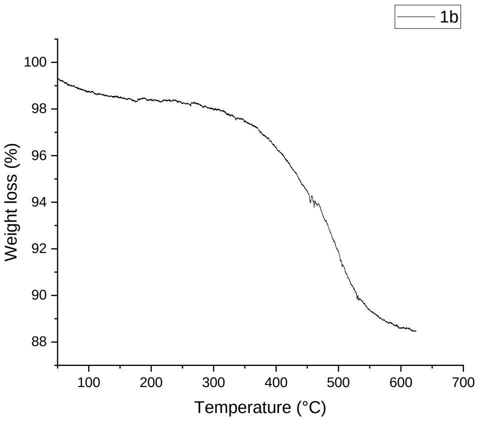

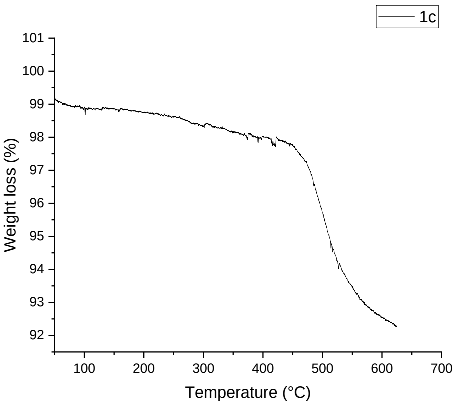

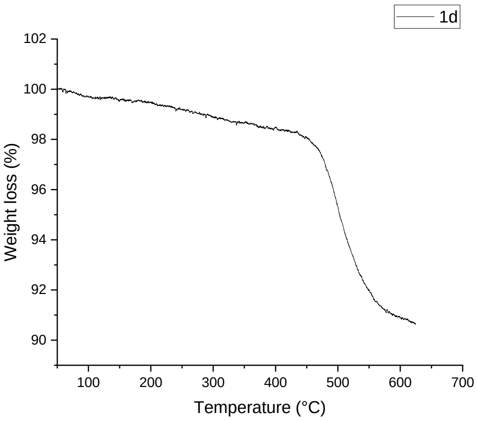

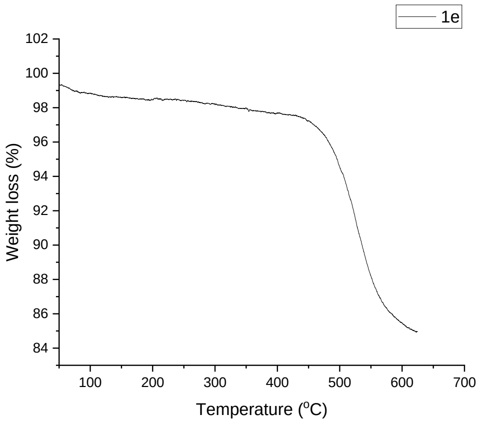

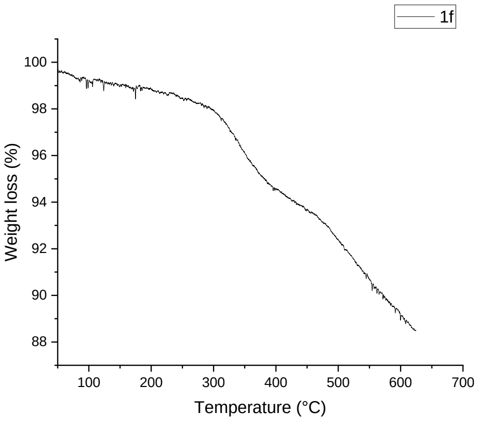

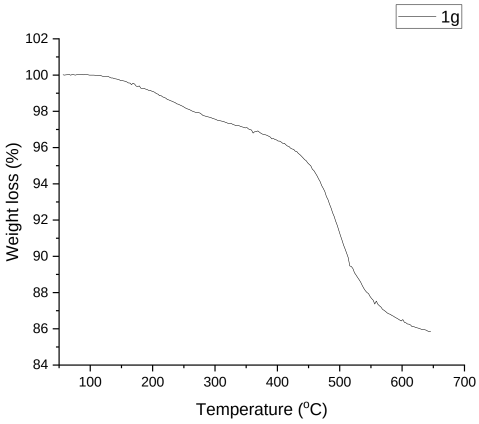
1g
Weight loss (%)
Temperature (°C)
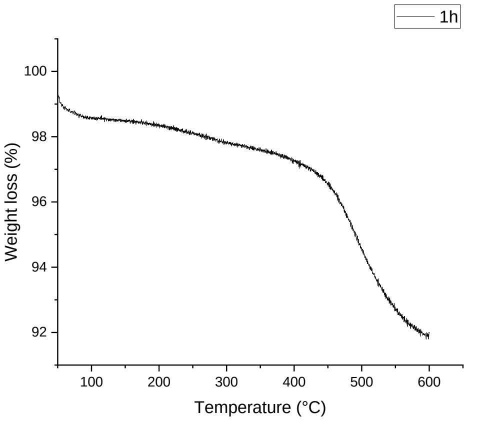
1h
Weight loss (%)
Temperature (°C)
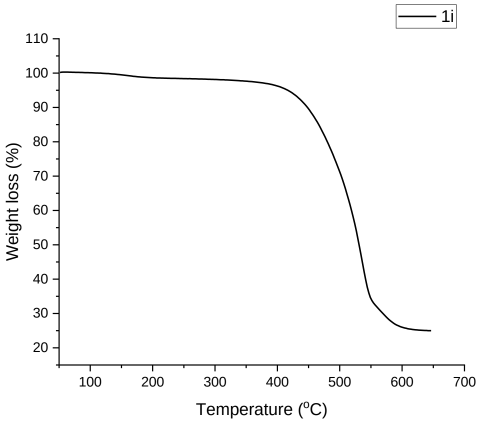
1i
Weight loss (%)
Temperature (°C)
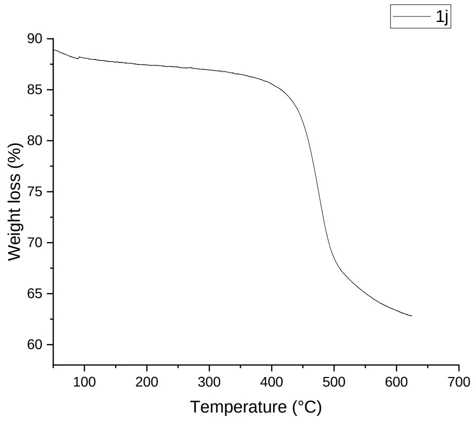
1j
Weight loss (%)
Temperature (°C)
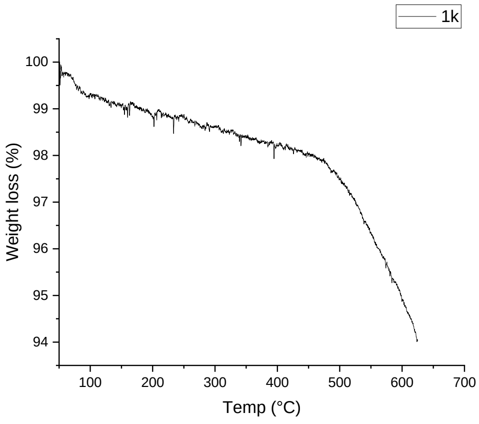
1k
Weight loss (%)
Temp (°C)
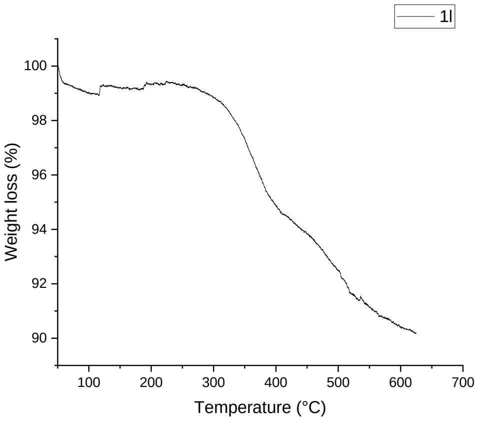
1l
Weight loss (%)
Temperature (°C)

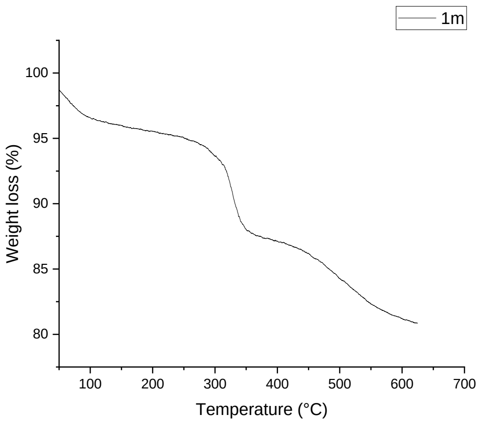
1m
Weight loss (%)
100
95
90
85
80
100 200 300 400 500 600 700
Temperature (°C)

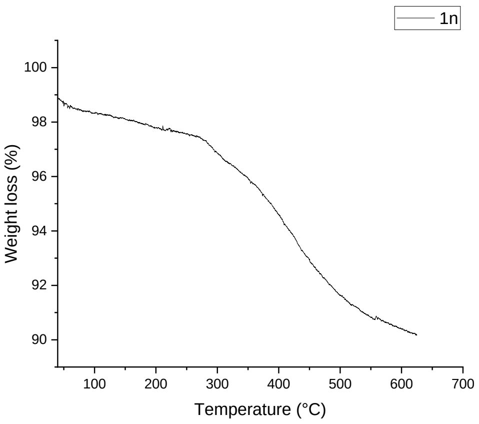
1n
Weight loss (%)
100
98
96
94
92
90
100 200 300 400 500 600 700
Temperature (°C)

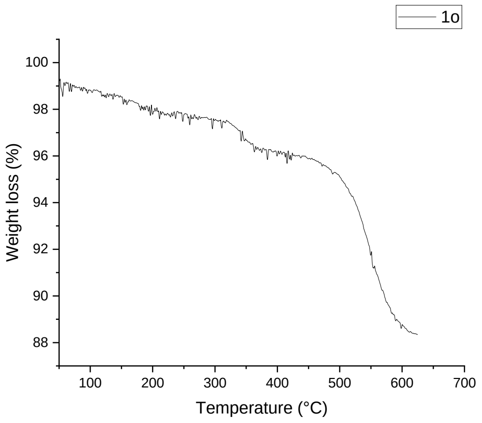
1o
Weight loss (%)
100
98
96
94
92
90
88
100 200 300 400 500 600 700
Temperature (°C)

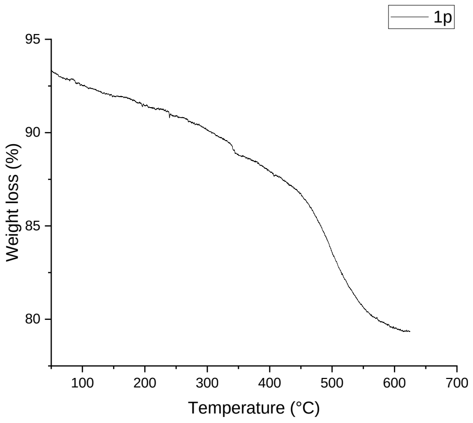
1p
Weight loss (%)
95
90
85
80
100 200 300 400 500 600 700
Temperature (°C)

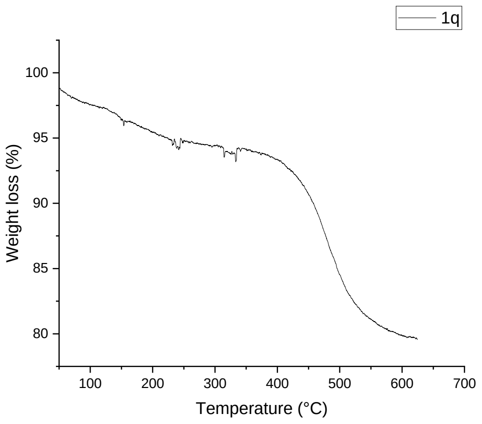
1q
Weight loss (%)
100
95
90
85
80
100 200 300 400 500 600 700
Temperature (°C)

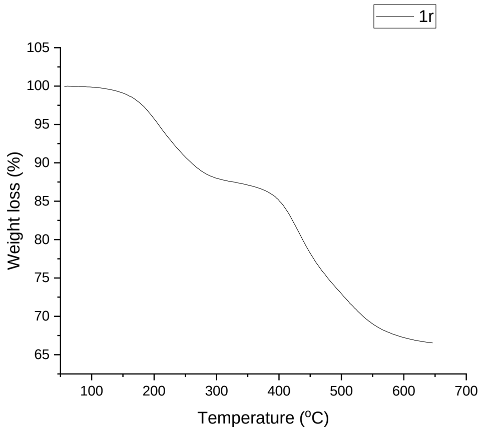
1r
Weight loss (%)
105
100
95
90
85
80
75
70
65
100 200 300 400 500 600 700
Temperature (ºC)

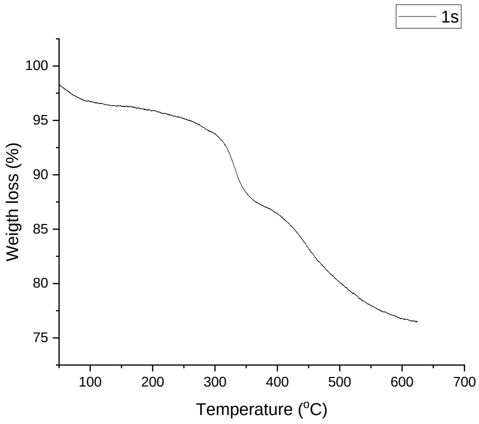
1s
Weigth loss (%)
Temperature (°C)
100
95
90
85
80
75
100 200 300 400 500 600 700

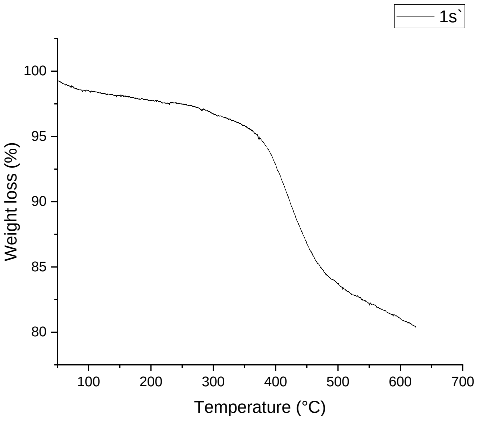
1s`
Weight loss (%)
Temperature (°C)
100
95
90
85
80
100 200 300 400 500 600 700

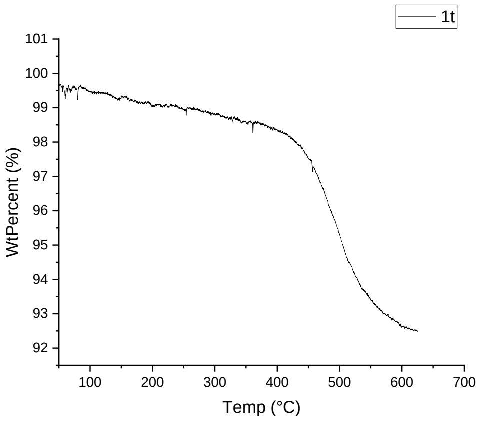
1t
WtPercent (%)
Temp (°C)
101
100
99
98
97
96
95
94
93
92
100 200 300 400 500 600 700

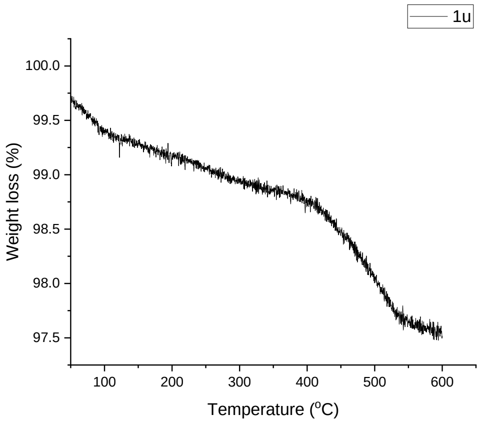
1u
Weight loss (%)
Temperature (°C)
100.0
99.5
99.0
98.5
98.0
97.5
100 200 300 400 500 600

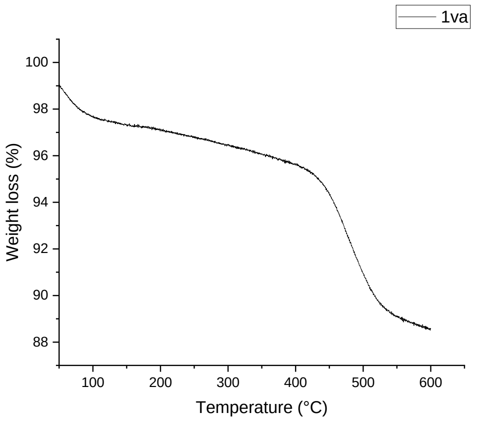
1va
Weight loss (%)
Temperature (°C)
100
98
96
94
92
90
88
100 200 300 400 500 600

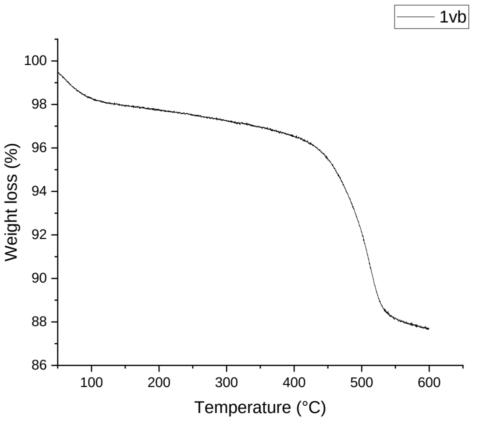
1vb
Weight loss (%)
Temperature (°C)
100
98
96
94
92
90
88
86
100 200 300 400 500 600

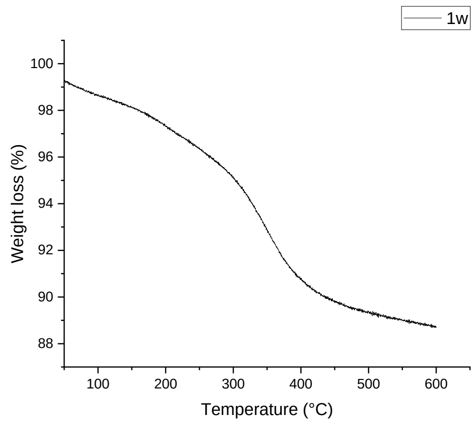
1w
Weight loss (%)
Temperature (°C)
100
98
96
94
92
90
88
100
200
300
400
500
600

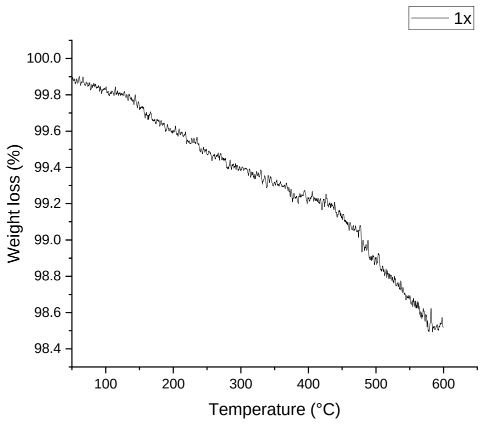
1x
Weight loss (%)
Temperature (°C)
100.0
99.8
99.6
99.4
99.2
99.0
98.8
98.6
98.4
100
200
300
400
500
600

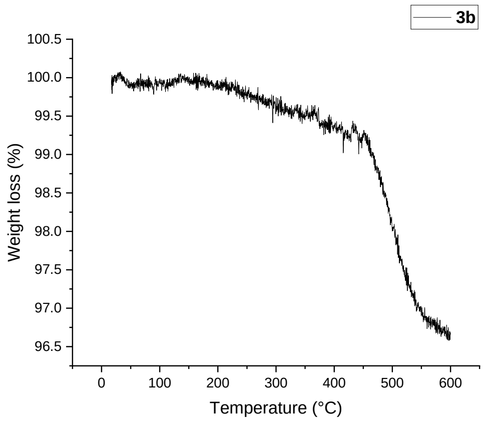
3b
Weight loss (%)
Temperature (°C)
100.5
100.0
99.5
99.0
98.5
98.0
97.5
97.0
96.5
0
100
200
300
400
500
600

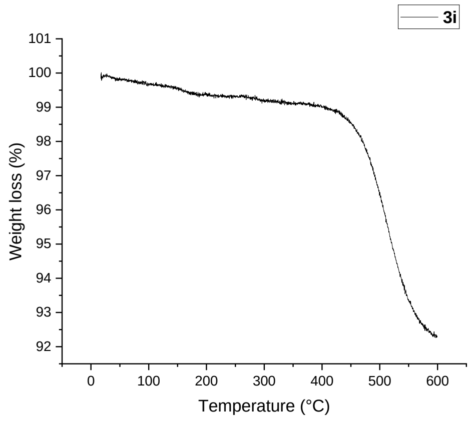
3i
Weight loss (%)
Temperature (°C)
101
100
99
98
97
96
95
94
93
92
0
100
200
300
400
500
600

S7. Analytic Data for Synthesized Compounds

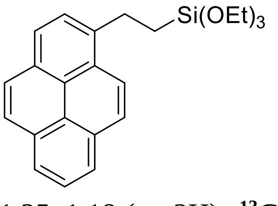

triethoxy(2-(pyren-1-yl)ethyl)silane (**E**): Yellow oil; **^{1}H NMR (400 MHz, $CDCl_3$)** δ 8.30 (d, *J* = 9.3 Hz, 1H), 8.16 (d, *J* = 5.4 Hz, 1H), 8.14 (d, *J* = 4.3 Hz, 1H), 8.12 (s, 1H), 8.10 (s, 1H), 8.07–7.95 (m, 3H), 7.91 (d, *J* = 7.8 Hz, 1H), 3.89 (q, *J* = 7.0 Hz, 6H), 3.51–3.41 (m, 2H), 1.28 (t, *J* = 7.0 Hz, 9H), 1.25–1.18 (m, 2H); **^{13}C NMR (101 MHz, $CDCl_3$)** δ 139.18, 131.55, 131.05, 129.84, 128.26, 127.64, 127.30, 126.57, 126.51, 125.84, 125.22, 125.19, 125.05, 124.89, 124.76, 123.42, 58.66, 26.78, 18.56, 13.44; **^{29}Si NMR (79 MHz, $CDCl_3$)** δ -45.64; **IR (neat)**: ν_{max} = 3048, 2965, 1603, 1186, 841, 726 cm^{-1}; **HRMS** m/z (EI) calc. for $C_{24}H_{29}O_3Si$ [M^+] 393.1881, found 393.1880; ***R**$_f$* 0.45 (5% ethyl acetate in petroleum ether).

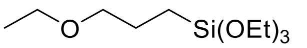

triethoxy(2-(pyren-1-yl)ethyl)silane (**F**): colorless liquid; **^{1}H NMR (400 MHz, $CDCl_3$)** δ 3.80 (q, *J* = 6.9 Hz, 6H), 3.45 (q, *J* = 7.0 Hz, 2H), 3.37 (t, *J* = 7.0 Hz, 2H), 1.73–1.62 (m, 2H), 1.20 (t, *J* = 7.0 Hz, 9H), 1.18 (t, *J* = 7.0 Hz, 3H), 0.70–0.54 (m, 2H); **^{13}C NMR (101 MHz, $CDCl_3$)** δ 73.14, 66.17, 58.56, 23.27, 18.51, 15.46, 6.72; **^{29}Si NMR (79 MHz, $CDCl_3$)** δ -44.49; **IR (neat)**: ν_{max} = 2972, 2882, 1389, 1100, 959, 798 cm^{-1}; **HRMS** m/z (EI) calc. for $C_{11}H_{26}NaO_4Si$ [M^+] 273.1498, found 273.1496; ***R**$_f$* 0.42 (10% ethyl acetate in petroleum ether).

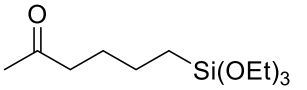

6-(triethoxysilyl)hexan-2-one (**G**): colorless liquid; **^{1}H NMR (400 MHz, $CDCl_3$)** δ 3.79 (q, *J* = 7.0 Hz, 6H), 2.40 (t, *J* = 7.4 Hz, 2H), 2.11 (s, 3H), 1.65–1.54 (m, 2H), 1.45–1.32 (m, 2H), 1.20 (t, *J* = 7.0 Hz, 9H), 0.66–0.55 (m, 2H); **^{13}C NMR (101 MHz, $CDCl_3$)** δ ^{13}C NMR (101 MHz, **$CDCl_3$**) δ 209.34, 58.51, 43.63, 30.00, 27.38, 22.67, 18.46, 10.47; **^{29}Si NMR (79 MHz, $CDCl_3$)** δ -44.80; **IR (neat)**: ν_{max} = 2975, 2880, 1716, 1357, 1079, 953, 781 cm^{-1}; **HRMS** m/z (EI) calc. for $C_{12}H_{26}NaO_4Si$ [M^+] 285.1498, found 285.1493; ***R**$_f$* 0.29 (10% ethyl acetate in petroleum ether).

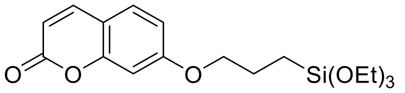

7-(3-(triethoxysilyl)propoxy)-2H-chromen-2-one (**J**): Yellow oil; **^{1}H NMR (400 MHz, $CDCl_3$)** δ 7.62 (d, *J* = 9.5 Hz, 1H), 7.34 (d, *J* = 8.5 Hz, 1H), 6.82 (dd, *J* = 8.5, 2.4 Hz, 1H), 6.79 (d, *J* = 2.5 Hz, 1H), 6.23 (d, *J* = 9.4 Hz, 1H), 3.99 (t, *J* = 6.6 Hz, 2H), 3.83 (q, *J* = 7.0 Hz, 6H), 1.99–1.86 (m, 2H), 1.22 (t, *J* = 7.0 Hz, 9H), 0.82–0.73 (m, 2H); **^{13}C NMR (101 MHz, $CDCl_3$)** δ 162.35, 161.23, 155.84, 143.53, 128.76, 112.87, 112.76, 112.33, 101.27, 70.38, 58.42, 22.58, 18.29, 6.45; **^{29}Si NMR (79 MHz, $CDCl_3$)** δ -45.42; **IR (neat)**: ν_{max} = 2972, 2884, 2275, 2392, 1123, 839 cm^{-1}; **HRMS** m/z (EI) calc. for $C_{18}H_{26}O_6SiNa$ [M^+] 389.1396, found 389.1391; ***R**$_f$* 0.50 (33% ethyl acetate in petroleum ether).

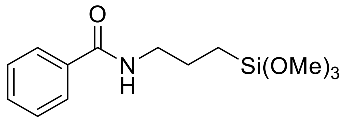

N-(3-(triethoxysilyl)propyl)benzamide (**S**): Pale yellow oil; **^{1}H NMR (400 MHz, $CDCl_3$)** δ 7.86–7.71 (m, 2H), 7.56–7.44 (m, 1H), 7.45–7.36 (m, 2H), 6.57 (s, 1H), 3.56 (s, 9H), 3.44 (q, *J* = 6.4 Hz, 2H), 1.86–1.68 (m, 2H), 0.83–0.58 (m, 2H); **^{13}C NMR (101 MHz, $CDCl_3$)** δ 167.70, 135.04, 131.43, 128.65, 127.03, 50.80, 42.43, 22.87, 6.75; **^{29}Si NMR (79 MHz, $CDCl_3$)** δ -41.67; **IR (neat)**: ν_{max} = 3321, 2942, 2840, 1639, 1540, 1294, 1078, 803 cm^{-1}; **HRMS** m/z (EI) calc. for $C_{16}H_{28}NO_4Si$ [M^+] 326.1783, found 326.1782; $\boldsymbol{R_f}$ 0.40 (5% Methanol in dichloromethane).

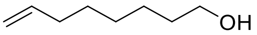

oct-7-en-1-ol, **2a**: colorless liquid; **^{1}H NMR (400 MHz, $CDCl_3$)** δ δ 5.81 (ddt, *J* = 16.9, 10.2, 6.7 Hz, 1H), 4.99 (ddt, *J* = 17.1, 2.2, 1.6 Hz, 1H), 4.93 (ddt, *J* = 10.2, 2.3, 1.2 Hz, 1H), 3.64 (t, *J* = 6.6 Hz, 2H), 2.16 – 1.89 (m, 2H), 1.62 – 1.57 (m, 2H), 1.42 – 1.32 (m, 6H); **^{13}C NMR (101 MHz, $CDCl_3$)** δ 139.28, 114.47, 63.27, 33.92, 32.95, 29.10, 29.07, 25.80; **IR (neat)**: ν_{max} = 3339, 2925, 2853, 1640, 1455, 1054, 909 cm^{-1}; $\boldsymbol{R_f}$ 0.32 (20% ethyl acetate in petroleum ether).

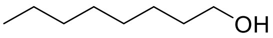

octan-1-ol, **2b**: colorless liquid; **^{1}H NMR (400 MHz, $CDCl_3$)** δ 3.64 (t, *J* = 6.6 Hz, 2H), 1.61 – 1.51 (m, 2H), 1.36 – 1.17 (m, 10H), 0.88 (t, *J* = 6.6 Hz, 3H); **^{13}C NMR (101 MHz, $CDCl_3$)** δ 63.34, 33.03, 32.03, 29.61, 29.49, 25.96, 22.87, 14.32; **IR (neat)**: ν_{max} = 3342, 2925, 2856, 1456, 1052, 724 cm^{-1}; $\boldsymbol{R_f}$ 0.32 (20% ethyl acetate in petroleum ether).

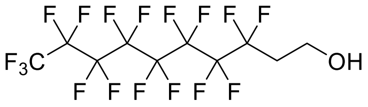

3,3,4,4,5,5,6,6,7,7,8,8,9,9,10,10,10-heptadecafluorodecan-1-ol, **2i**: white solid; **^{1}H NMR (400 MHz, $CDCl_3$)** δ 4.00 (q, *J* = 6.2 Hz, 2H), 2.49–2.29 (m, 2H), 1.67 (t, *J* = 5.8 Hz, 1H); **^{13}C NMR (101 MHz, $CDCl_3$)** δ 55.51 (t, J_{C-F} = 4.8 Hz), 34.11 (t, J_{C-F} = 21.3 Hz) (fluorinated carbon peaks are omitted because their intensity is reduced by splitting); **^{19}F NMR (376 MHz, $CDCl_3$)** δ -80.72 (t, *J* = 10.1 Hz), -113.40 (p, *J* = 17.0 Hz), -121.64, -121.86 (2 x CF_2), -122.66, -123.64, -126.07; **IR (neat)**: ν_{max} = 3340, 1207, 1147, 911, 742 cm^{-1}; $\boldsymbol{R_f}$ 0.32 (20% ethyl acetate in petroleum ether).

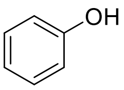

phenol, **2k**: colorless solid; **^{1}H NMR (400 MHz, $CDCl_3$)** δ 7.31–7.19 (m, 2H), 6.94 (tt, *J* = 7.3, 1.1 Hz, 1H), 6.89–6.79 (m, 2H), 4.80 (s, 1H); **^{13}C NMR (101 MHz, $CDCl_3$)** δ 155.67, 129.89, 121.01, 115.48; **IR (neat)**: ν_{max} = 3305, 2925, 2852, 1594, 1473,1237, 752, 690 cm^{-1}; $\boldsymbol{R_f}$ 0.50 (20% ethyl acetate in petroleum ether).

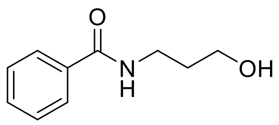

N-(3-hydroxypropyl)benzamide, **2s**: white solid; **^{1}H NMR (400 MHz, $CDCl_3$)** δ 7.85–7.74 (m, 2H), 7.54–7.48 (m, 1H), 7.47–7.40 (m, 2H), 6.64 (s, 1H), 3.73 (t, *J* = 5.6 Hz, 2H), 3.65 (q, *J* = 6.1 Hz, 2H), 3.12 (s, 1H), 1.87–1.75

(m, 2H); **^{13}C NMR (101 MHz, $CDCl_3$)** δ 168.74, 134.39, 131.84, 128.84, 127.11, 60.02, 37.34, 32.44; **IR (neat)**: ν_{max} = 3289, 2927, 2856, 1642, 1548, 1306, 1056, 693 cm^{-1}; ***R_f*** 0.20 (5% Methanol in dichloromethane).

S8. NaOH digestion of nanoparticles 1a

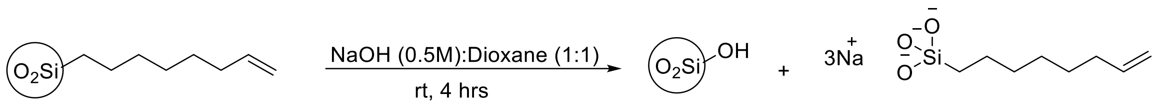

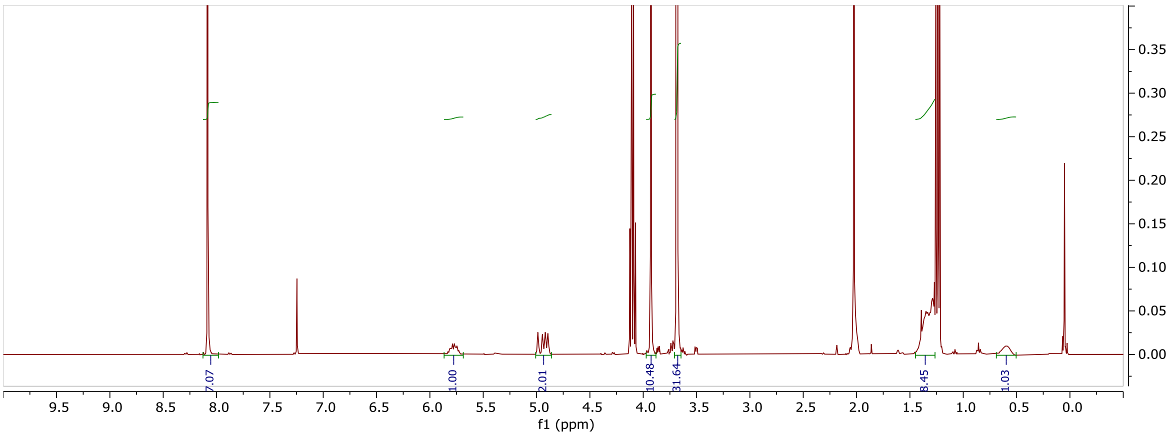

Digestion of nanoparticles **1a** by NaOH followed by NMR analysis in dioxane using dimethyl terephthalate as internal standard.

S9. Quantitative detection of octanol by ^{1}H NMR and GC-MS

To confirm quantitative detection of dissociated species, a sample of authentic octanol was subjected to the dissociation conditions and subsequently analysed. n-Octan-1-ol (9.10 mg) was added to a solution of diethyl ether (1 mL), $KHCO_3$ (31.2 mg), Bu_4NF (312 µl), H_2O_2 (53 µL) and stirred. The solution was filtered through a silica plug with a diethyl ether/ethyl acetate (3:1) mixture. The filtrate was collected and evaporated to dryness. The sample was quantified using ^{1}H NMR (dimethylterepthalate as the internal standard, 10 mg). The same ^{1}H NMR sample was evaporated to dryness before derivatisation. Silylation was performed by the addition of N,O-bis(trimethylsilyl)trifluoroacetamide (BSTFA) containing 1 % trimethylchlorosilane (TMCS; 100 µL) and pyridine (20 µL). The solution was heated at 100 °C for 30 mins. The mixture was first quantified by ^{1}H NMR. Then evaporated to dryness before being diluted in DCM (10 mL) and quantified by GC-MS.

**Initial octanol mass /mg**	**Mass after filtration (NMR) /mg**	**Mass after silylation (NMR) /mg**	**Mass after silylation (GC-MS) /mg**
9.10	8.98	8.79	9.45

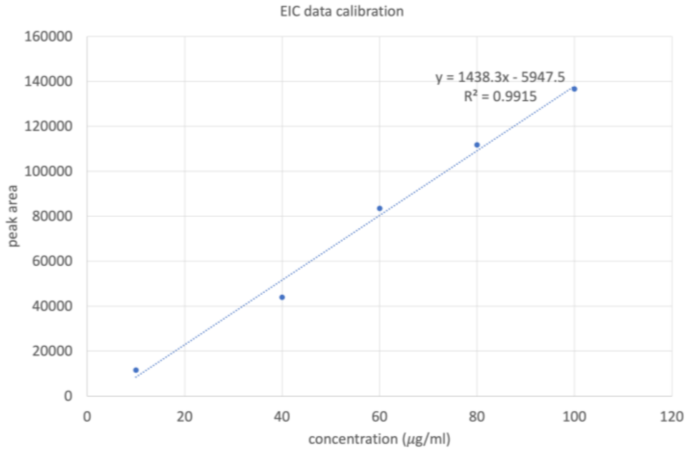

GC-MS calibration of octanol **2b** after silylation.

S10. GC-MS data of monolayer 1b digestion from Petri dish

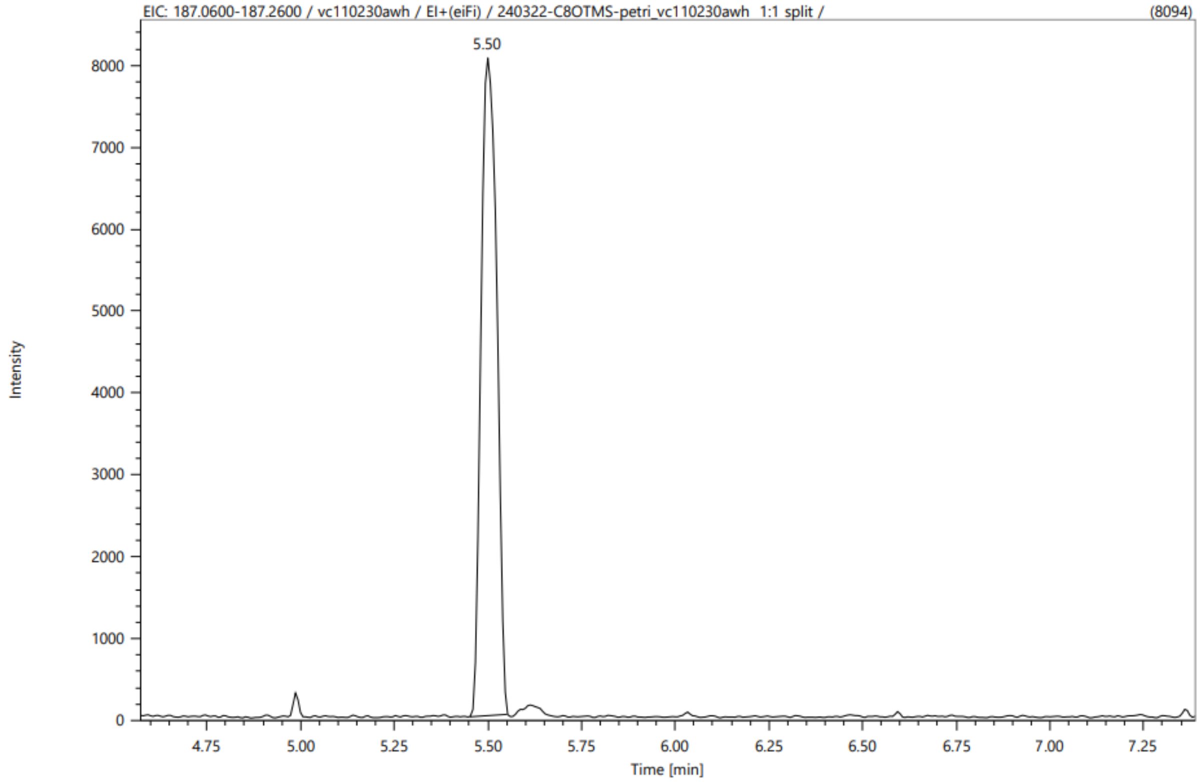

GC-MS (electron impact): extracted ion chromatogram of octanol **2b** following digestion from Petri dish and silylation.

S11. References

(S1) Feng, X.; Fryxell, G. E.; Wang, L.-Q.; Kim, A. Y.; Liu, J.; Kemner, K. M. Functionalized Monolayers on Ordered Mesoporous Supports. *Science* **1997**, *276* (5314), 923-926.

(S2) Tomashchuk, I.; Kostenko, L.; Jouvard, J.-M.; Lavisse, L.; del Carmen Marco de Lucas, M. Covalent grafting of alkyl chains on laser-treated titanium surfaces through silanization and phosphonation reactions. *Appl. Surf. Sci.* **2023**, *609*, 155390.

(S3) Malmström, E.; Miller, R. D.; Hawker, C. J. Development of a new class of rate-accelerating additives for nitroxide-mediated 'living' free radical polymerization. *Tetrahedron* **1997**, *53* (45), 15225-15236.

(S4) Talebzadeh, S.; Queffélec, C.; Knight, D. A. Surface modification of plasmonic noble metal–metal oxide core–shell nanoparticles. *Nanoscale Adv.* **2019**, *1* (12), 4578-4591.

(S5) Karstedt, B. D. Platinum complexes of unsaturated siloxanes and platinum containing organopolysiloxanes. **1973**, US Patent 3775452.

(S6) Kong, C. J.; Gilliland, S. E.; Clark, B. R.; Gupton, B. F. Highly-active, graphene-supported platinum catalyst for the solventless hydrosilylation of olefins. *Chem. Comm.* **2018**, *54* (95), 13343-13346

S12. NMR Spectra (^{1}H NMR, ^{13}C NMR and ^{19}F NMR)

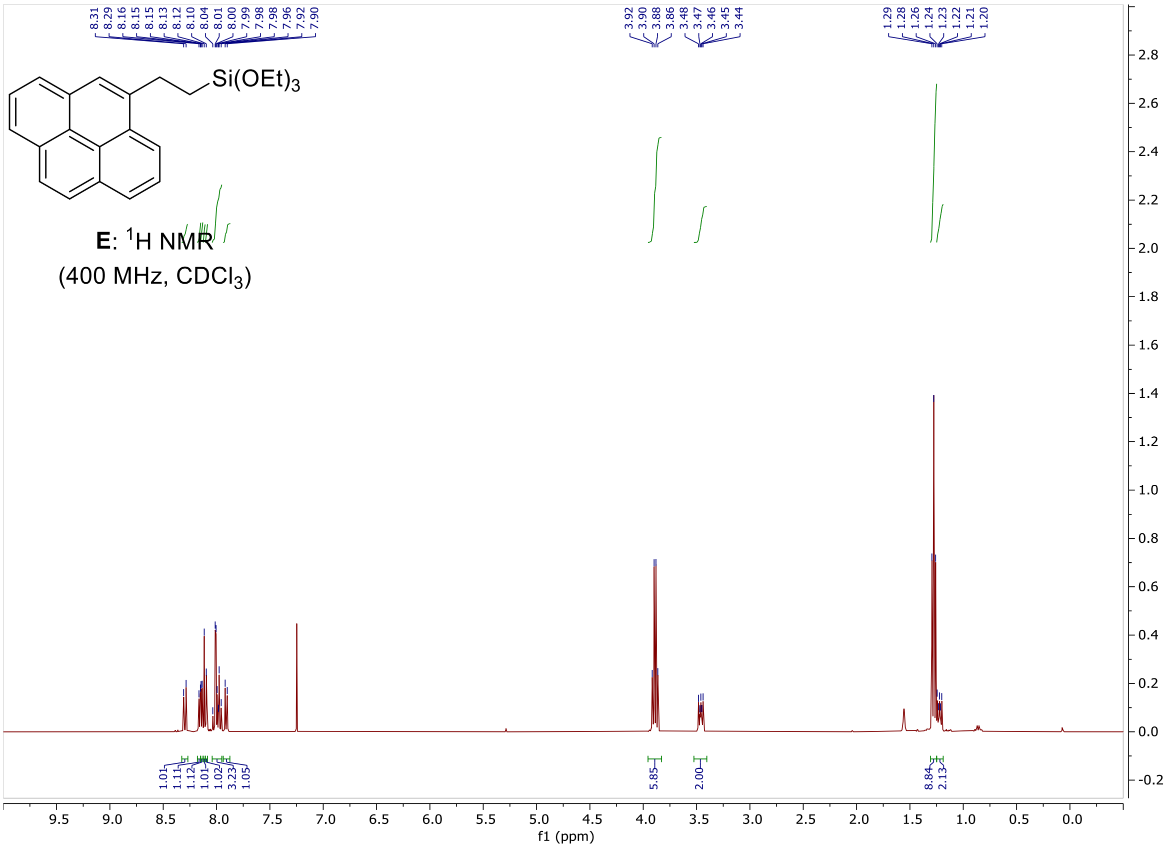

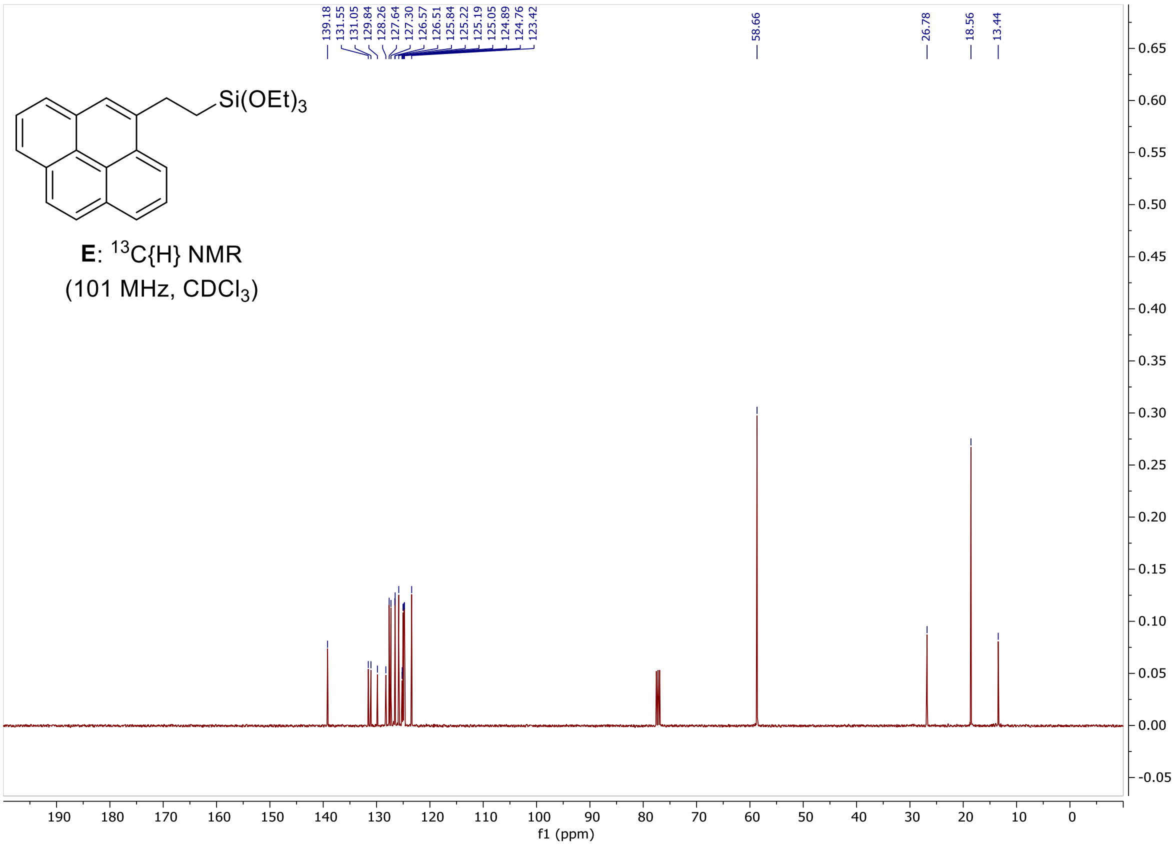

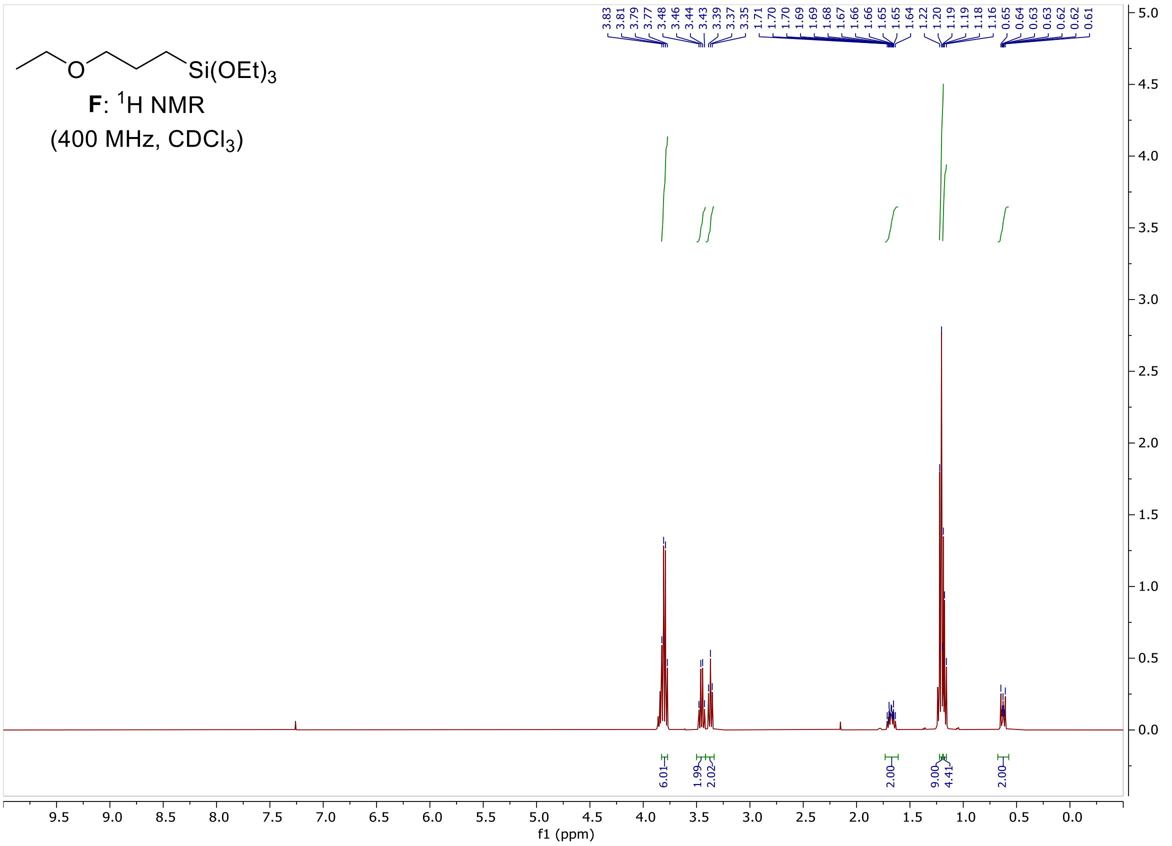
Si(OEt)3
F: 1H NMR
(400 MHz, CDCl3)
3.83 3.81 3.79 3.77 3.48 3.46 3.44 3.43 3.39 3.37 3.35 1.71 1.70 1.69 1.69 1.68 1.67 1.66 1.66 1.65 1.65 1.64 1.22 1.20 1.19 1.19 1.18 1.16 0.65 0.64 0.63 0.63 0.62 0.62 0.61
6.01 1.99 2.02 2.00 9.00 4.41 2.00
f1 (ppm)

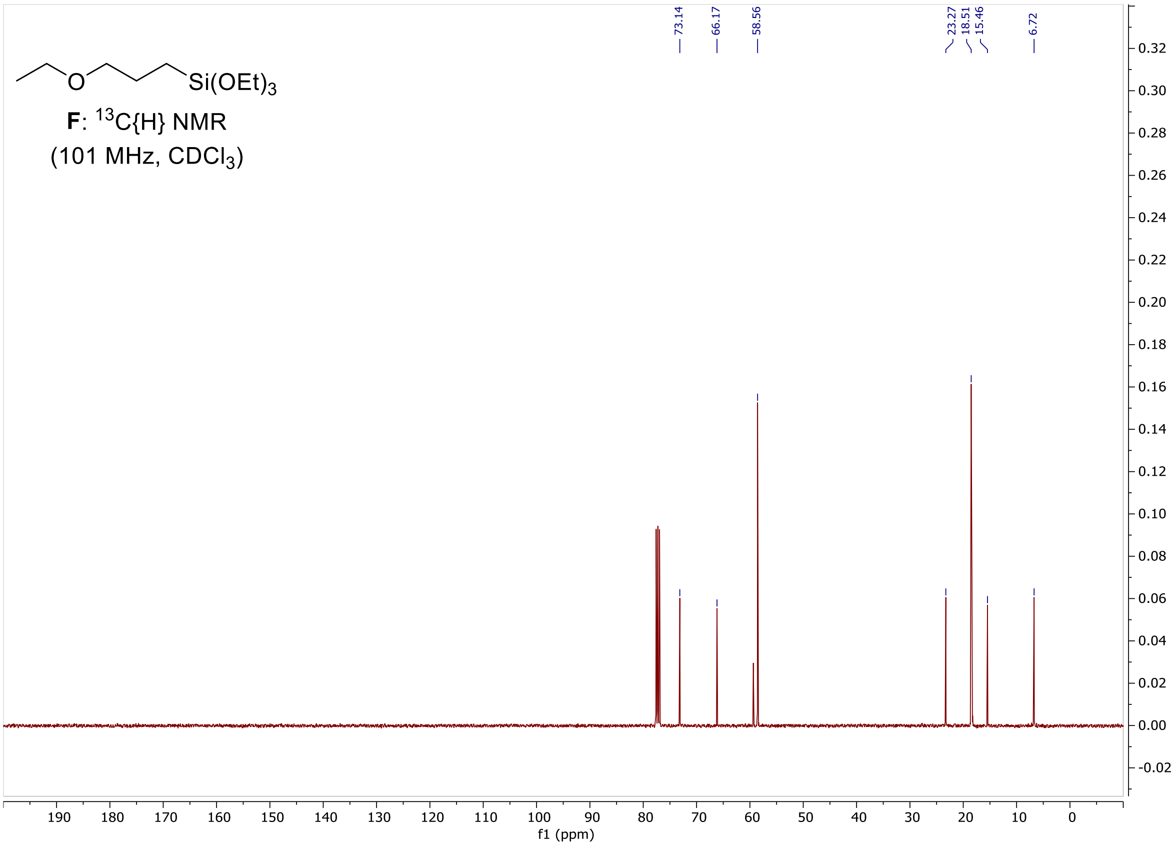
Si(OEt)3
F: 13C{H} NMR
(101 MHz, CDCl3)
73.14 66.17 58.56 23.27 18.51 15.46 6.72
f1 (ppm)

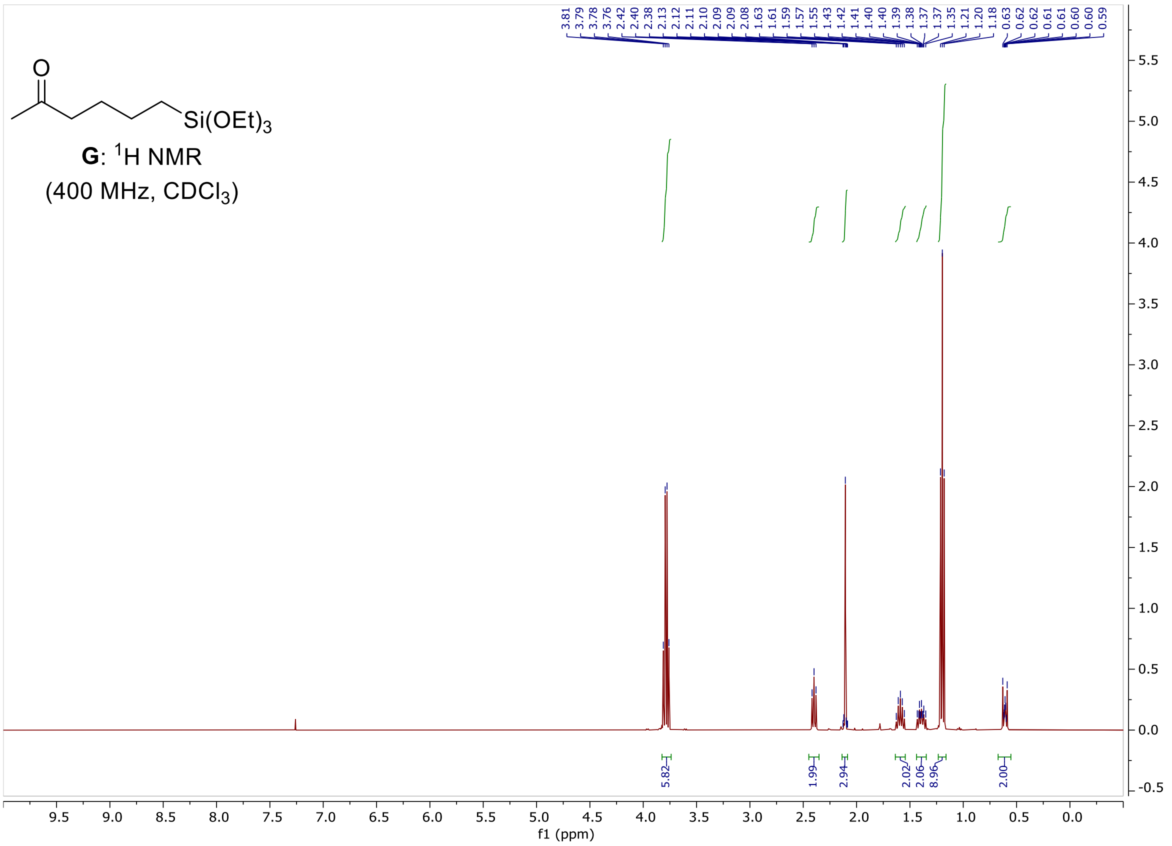
O
Si(OEt)3
G: 1H NMR
(400 MHz, CDCl3)
5.82
1.99
2.94
2.02
2.06
8.96
2.00
f1 (ppm)

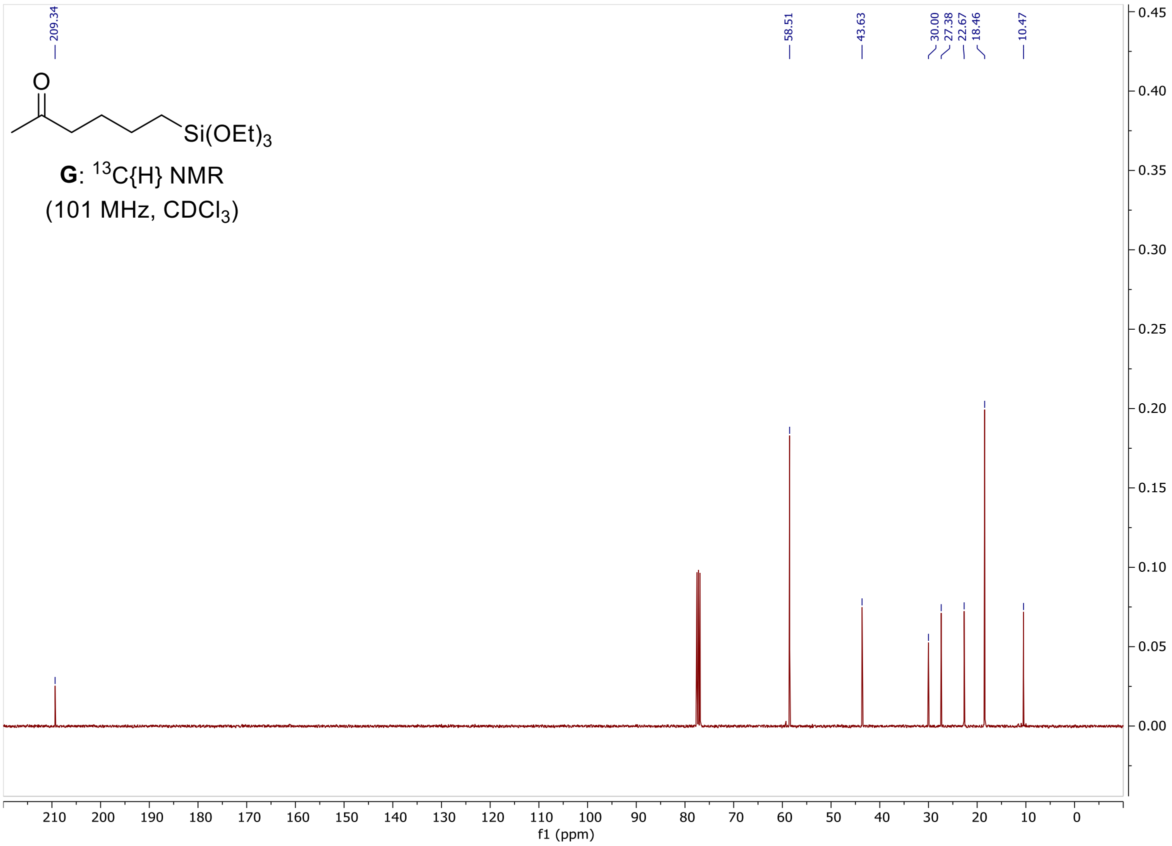
209.34
58.51
43.63
30.00
27.38
22.67
18.46
10.47
O
Si(OEt)3
G: 13C{H} NMR
(101 MHz, CDCl3)
f1 (ppm)

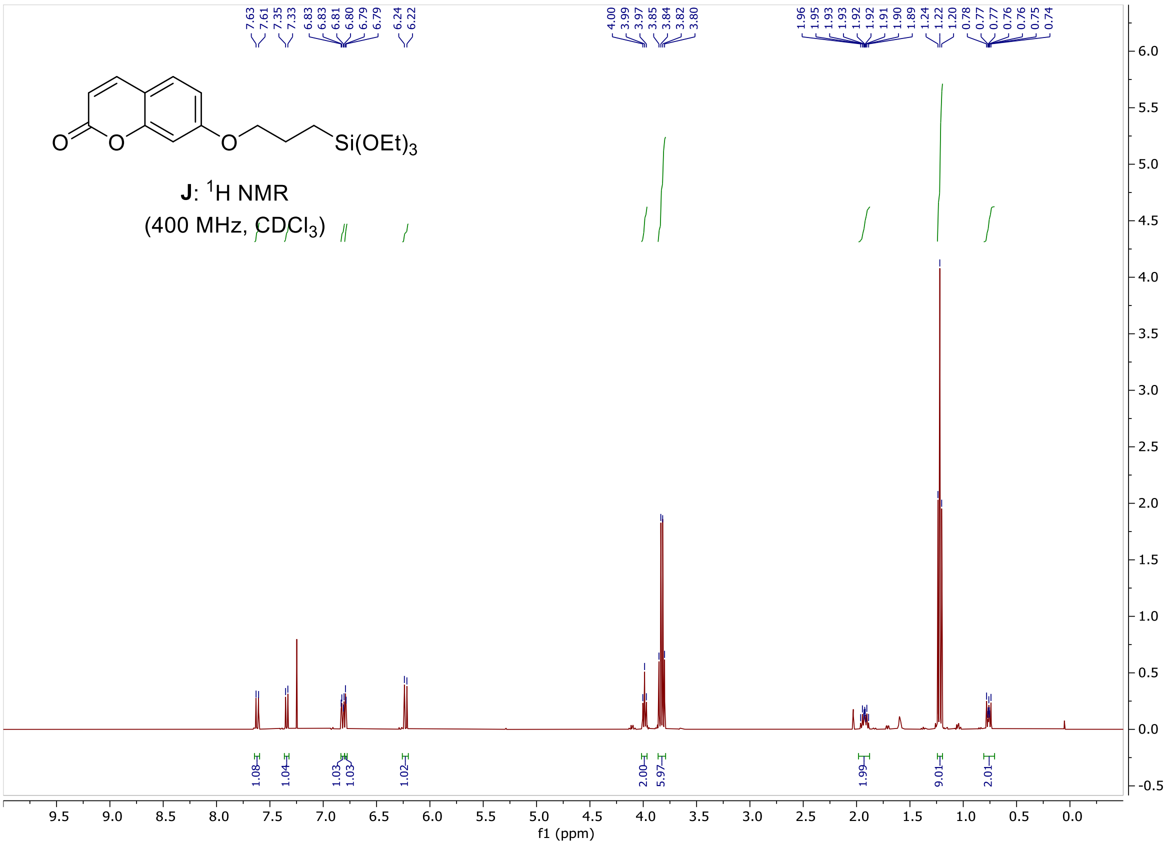
J: ^{1}H NMR
(400 MHz, $CDCl_3$)
Si(OEt)3
f1 (ppm)

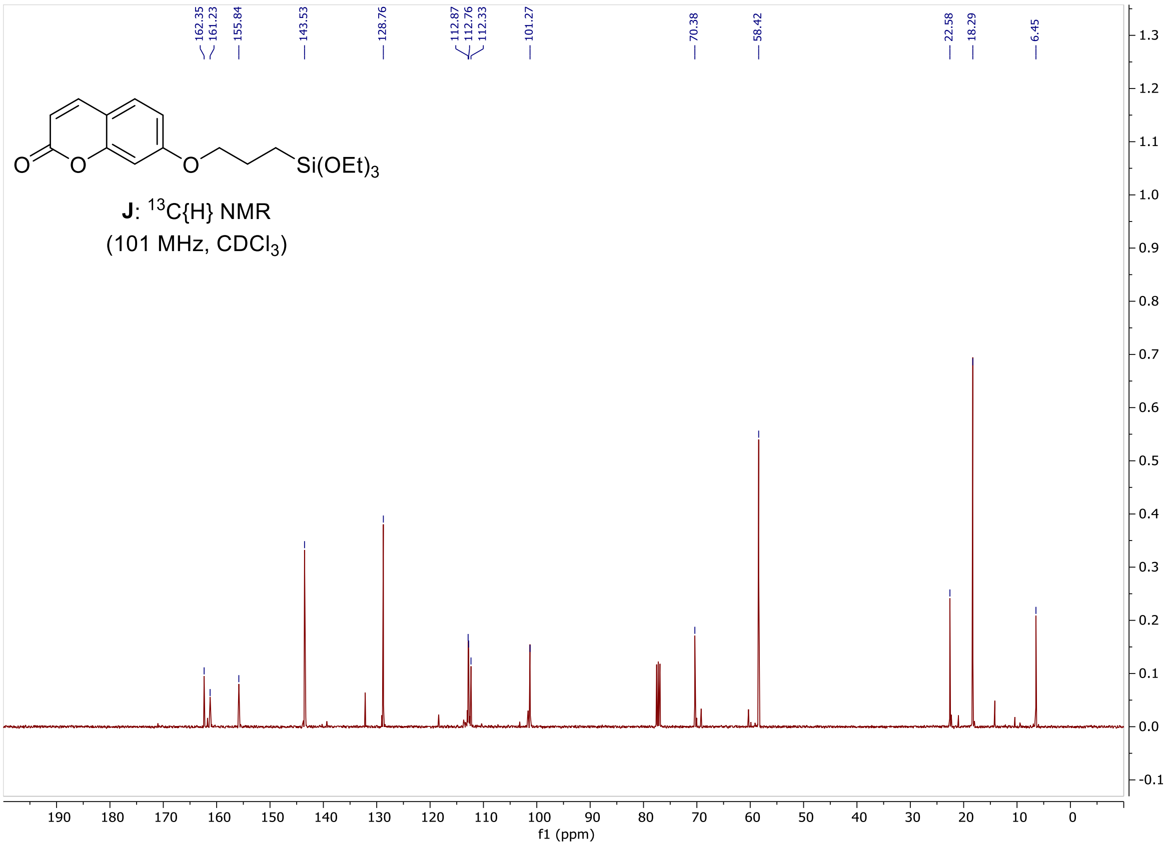
J: ^{13}C{H} NMR
(101 MHz, $CDCl_3$)
Si(OEt)3
162.35
161.23
155.84
143.53
128.76
112.87
112.76
112.33
101.27
70.38
58.42
22.58
18.29
6.45
f1 (ppm)

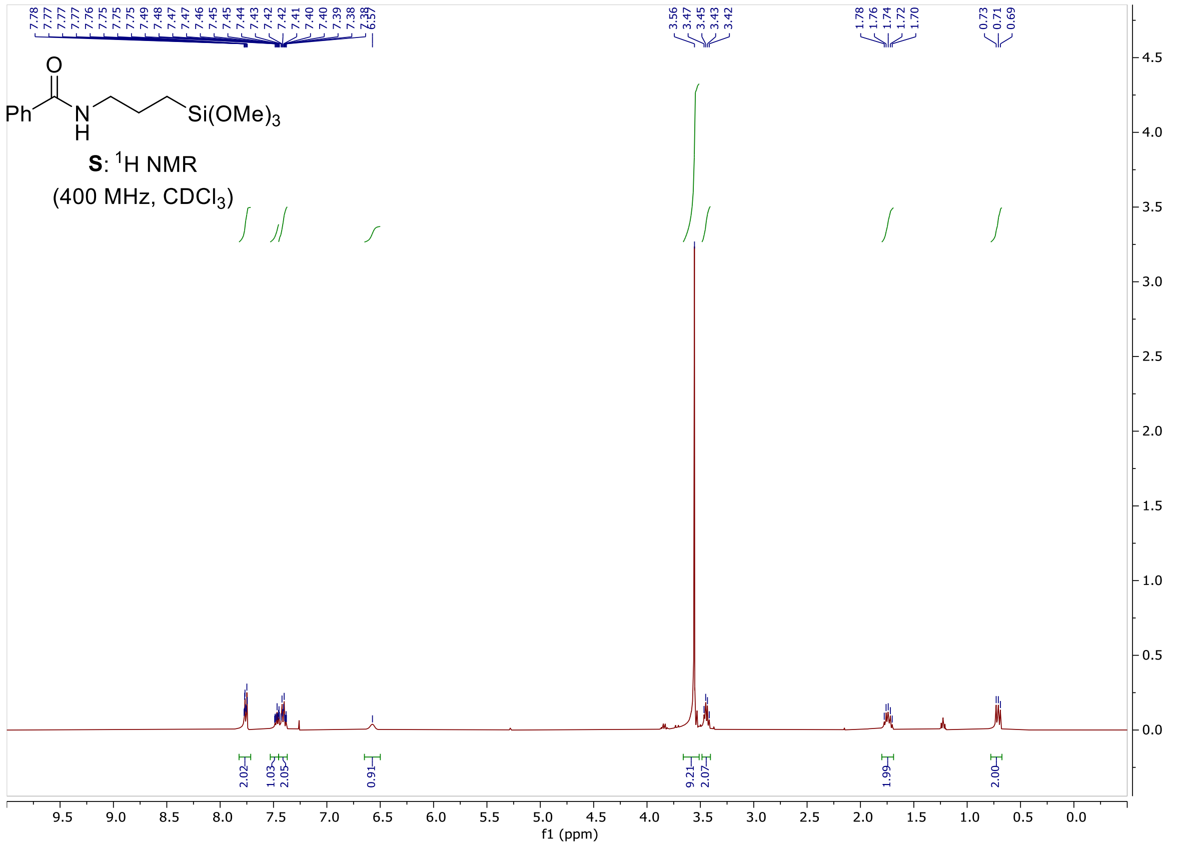
Ph
O
N
H
Si(OMe)3
S: 1H NMR
(400 MHz, CDCl3)
7.78 7.77 7.77 7.77 7.76 7.75 7.75 7.75 7.49 7.48 7.47 7.47 7.46 7.45 7.45 7.44 7.43 7.42 7.42 7.41 7.40 7.40 7.39 7.38
3.56 3.47 3.45 3.43 3.42
1.78 1.76 1.74 1.72 1.70
0.73 0.71 0.69
2.02 1.03 2.05 0.91 9.21 2.07 1.99 2.00
f1 (ppm)

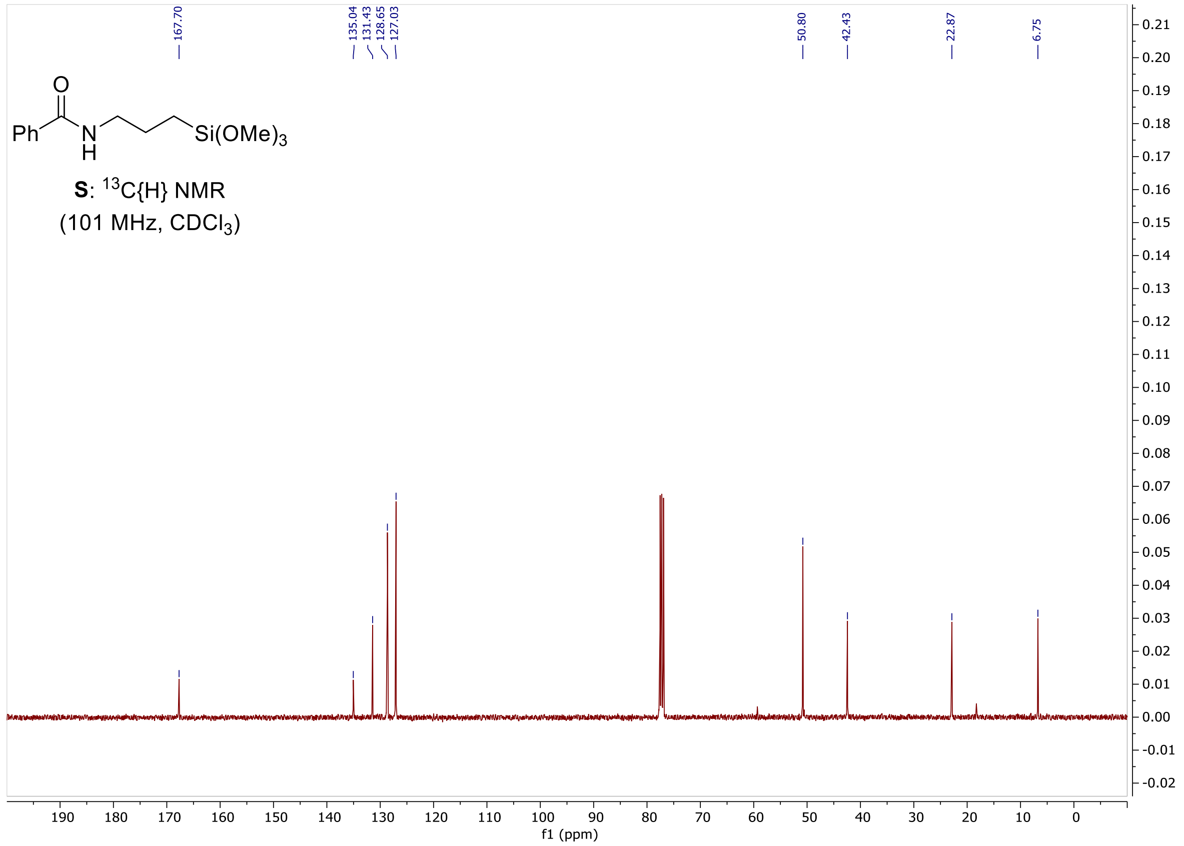
Ph
O
N
H
Si(OMe)3
S: 13C{H} NMR
(101 MHz, CDCl3)
167.70
135.04 131.43 128.65 127.03
50.80
42.43
22.87
6.75
f1 (ppm)

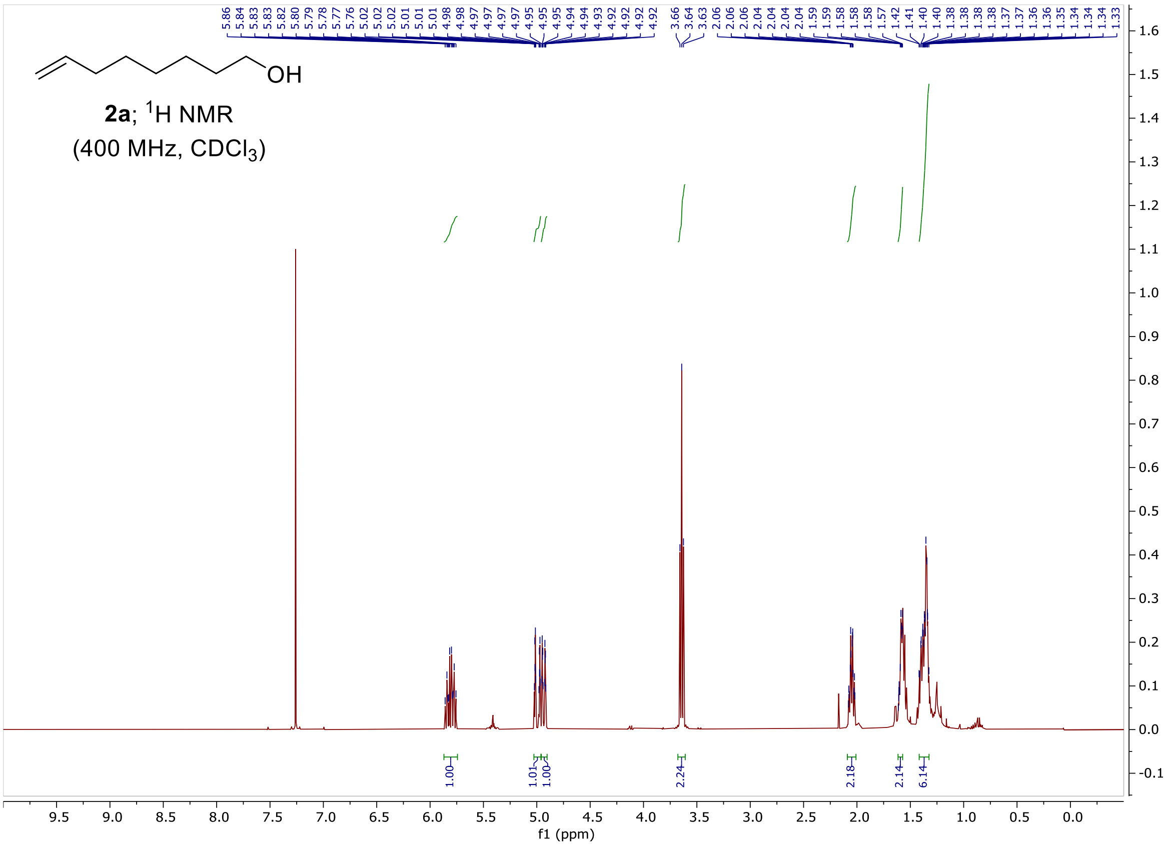
OH
2a; 1H NMR
(400 MHz, CDCl3)
f1 (ppm)

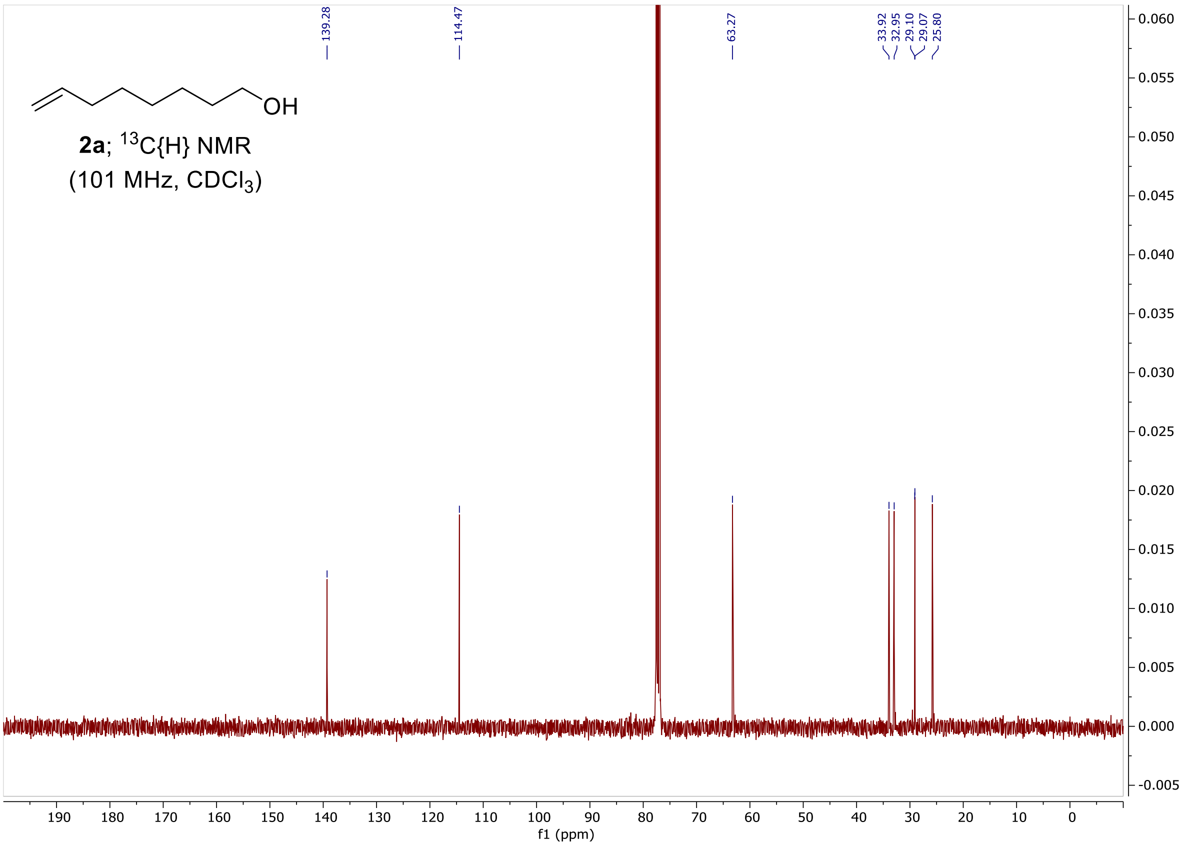
OH
2a; 13C{H} NMR
(101 MHz, CDCl3)
139.28
114.47
63.27
33.92
32.95
29.10
29.07
25.80
f1 (ppm)

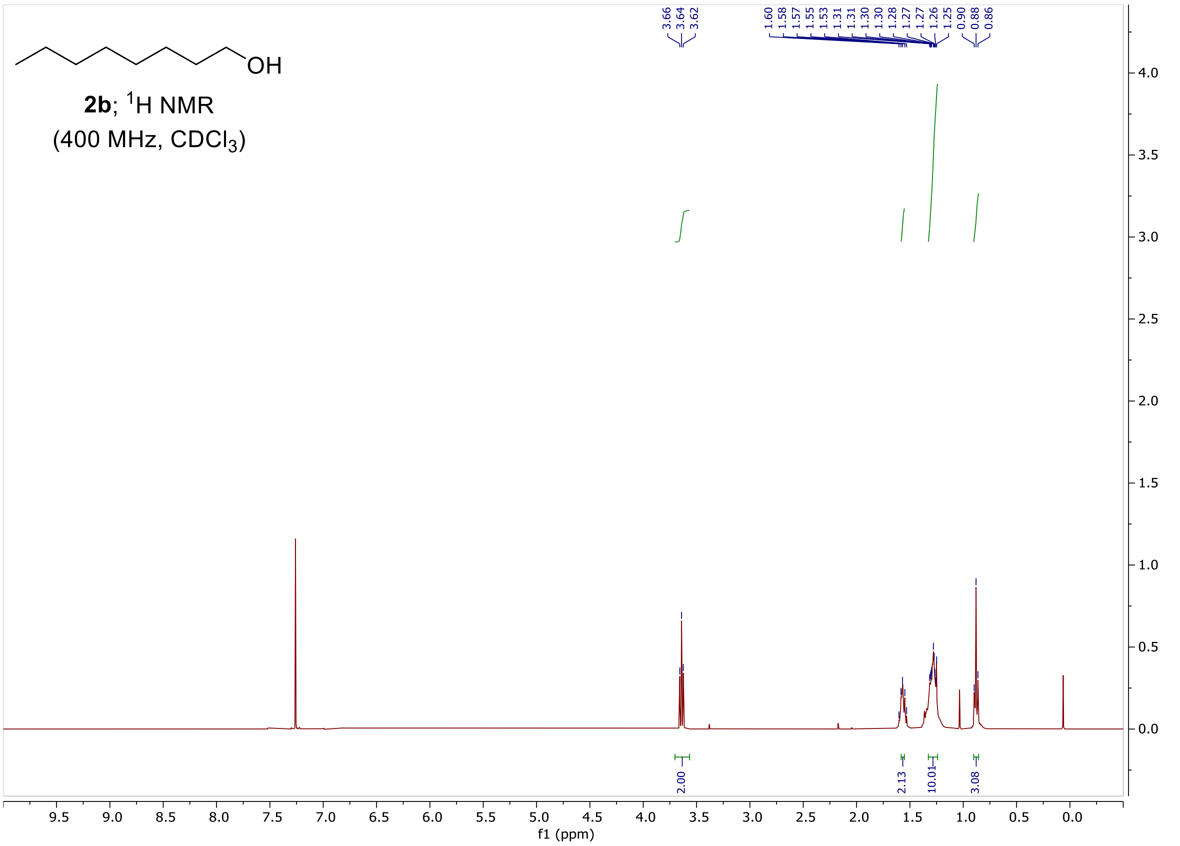

OH
2b; 1H NMR
(400 MHz, CDCl3)
3.66
3.64
3.62
1.60
1.58
1.57
1.55
1.53
1.31
1.31
1.30
1.30
1.28
1.27
1.27
1.26
1.25
0.90
0.88
0.86
2.00
2.13
10.01
3.08
f1 (ppm)

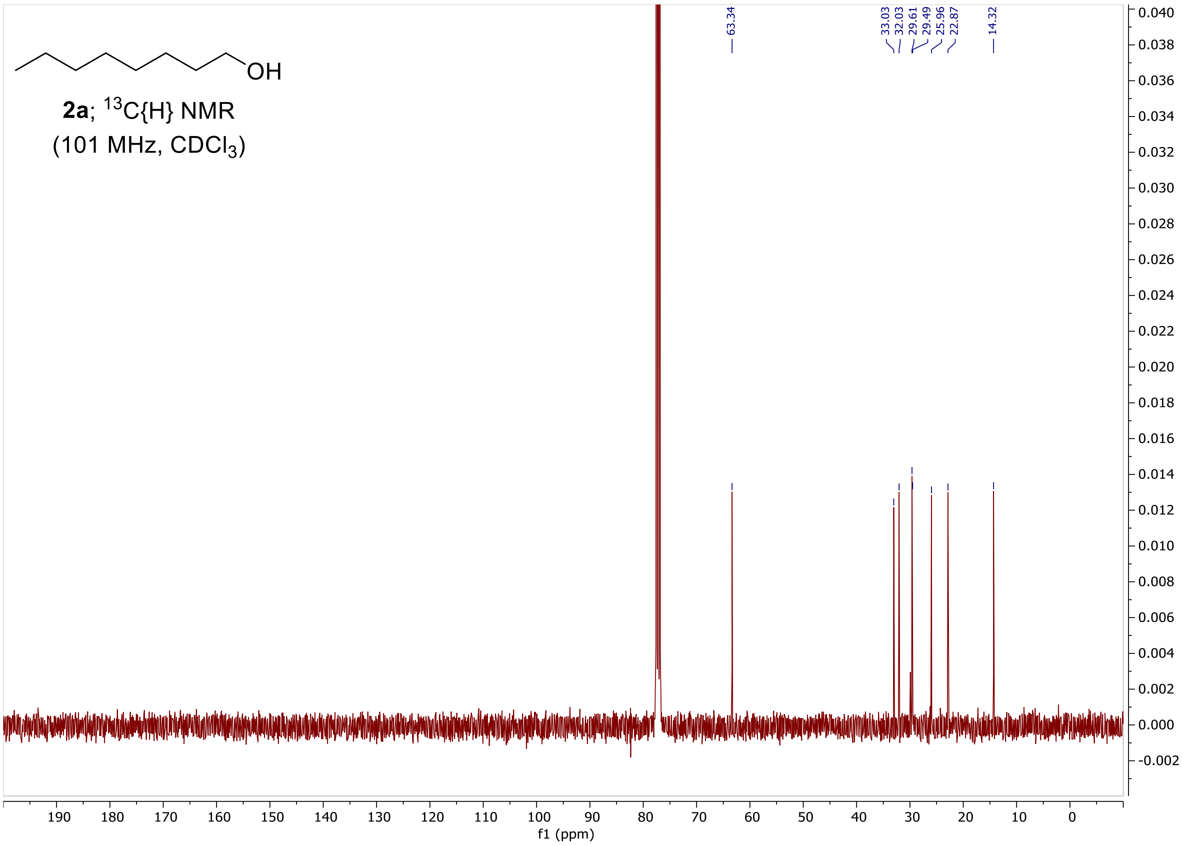

OH
2a; 13C{H} NMR
(101 MHz, CDCl3)
63.34
33.03
32.03
29.61
29.49
25.96
22.87
14.32
f1 (ppm)

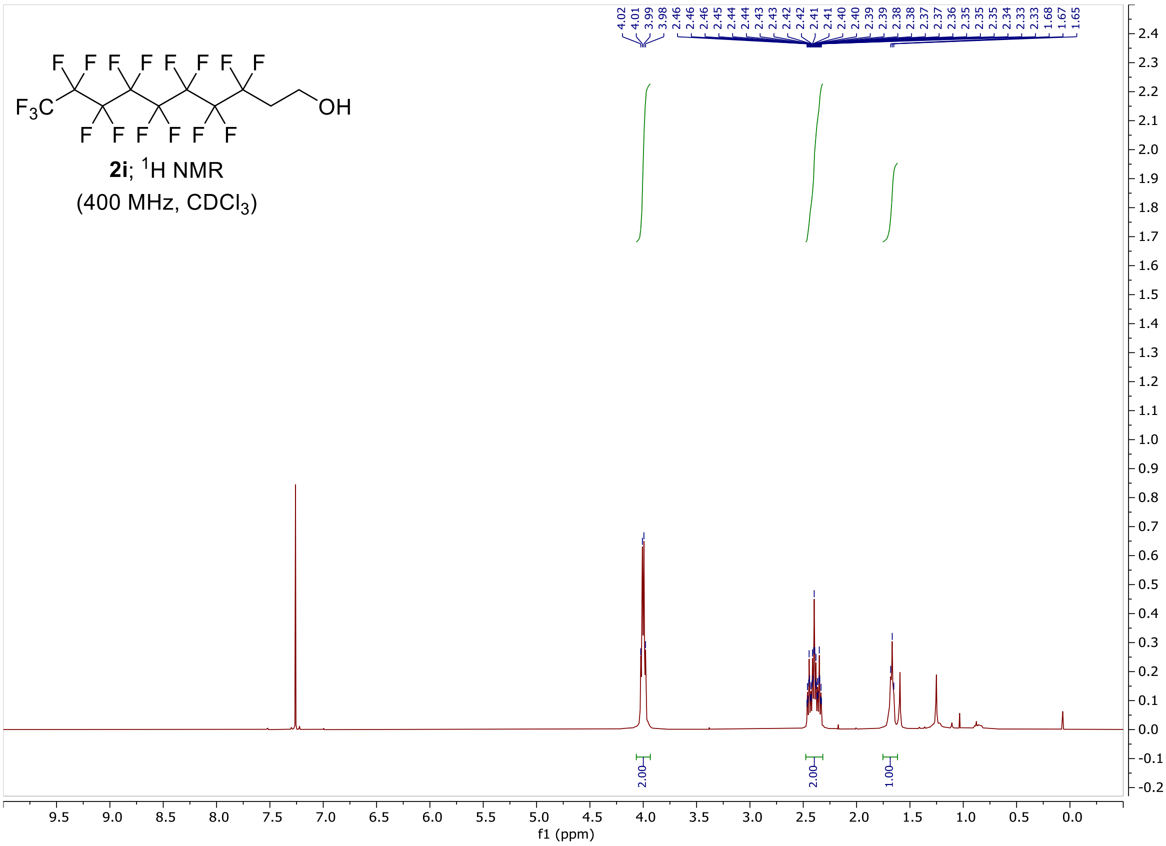
F F F F F F F F
F3C
OH
F F F F F F
2i; 1H NMR
(400 MHz, CDCl3)
4.02
4.01
3.99
3.98
2.46
2.46
2.45
2.44
2.44
2.43
2.43
2.42
2.42
2.41
2.41
2.40
2.40
2.39
2.39
2.38
2.38
2.37
2.37
2.36
2.35
2.35
2.35
2.34
2.33
2.33
1.68
1.67
1.65
2.00
2.00
1.00
f1 (ppm)

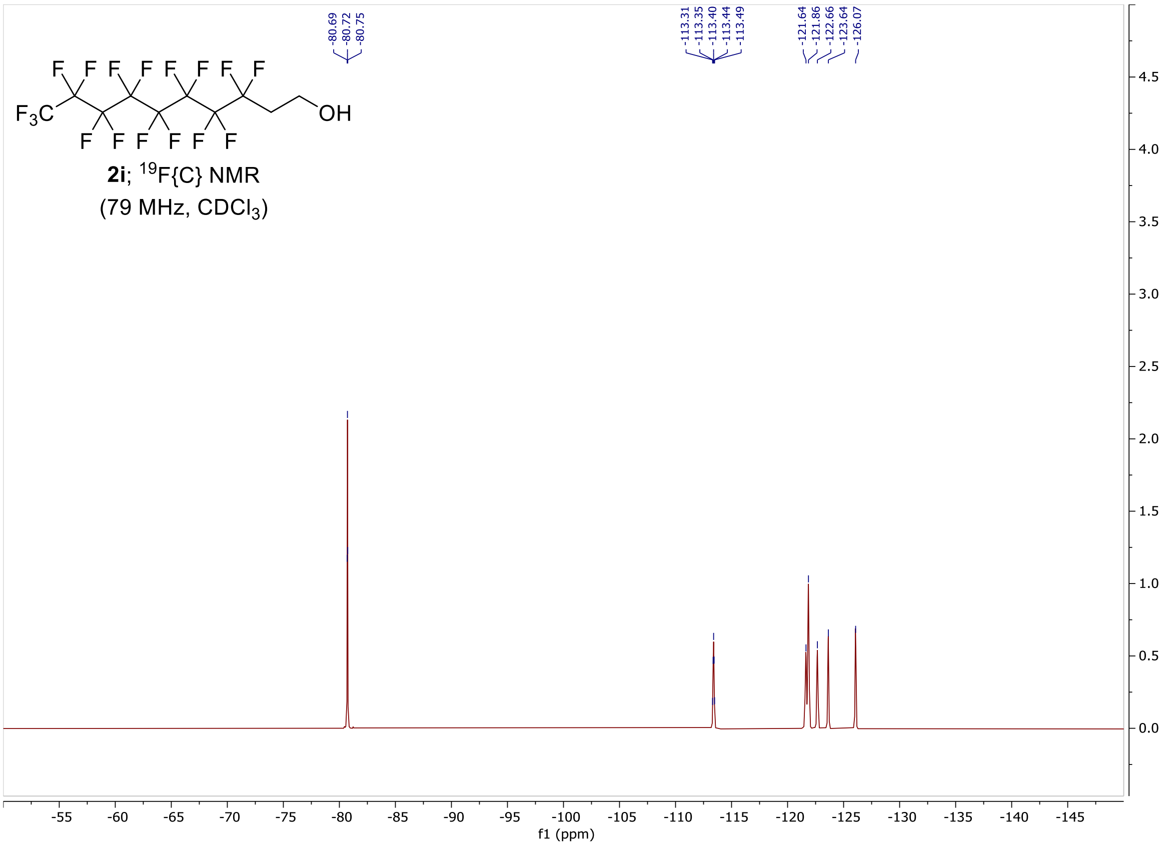
F F F F F F F F
F3C
OH
F F F F F F
2i; 19F{C} NMR
(79 MHz, CDCl3)
-80.69
-80.72
-80.75
-113.31
-113.35
-113.40
-113.44
-113.49
-121.64
-121.86
-122.66
-123.64
-126.07
f1 (ppm)

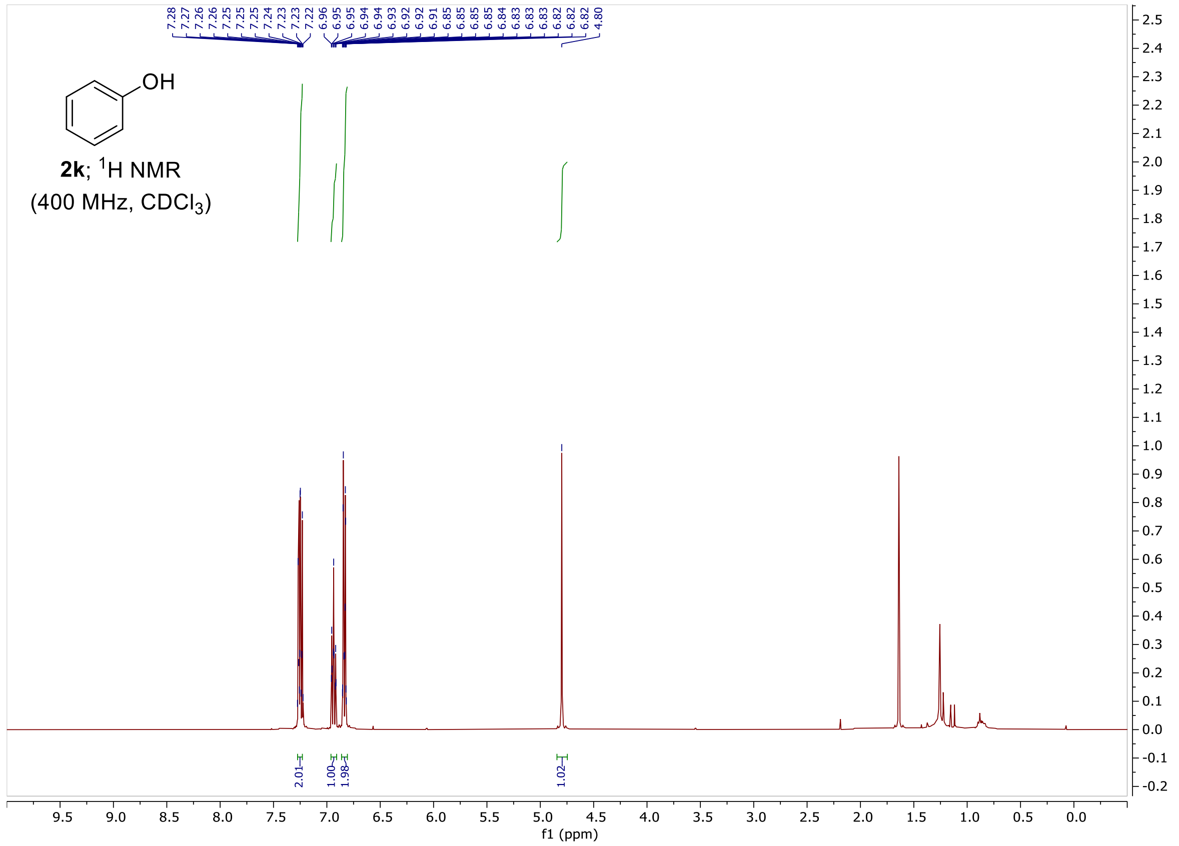
OH
2k; 1H NMR
(400 MHz, CDCl3)
7.28 7.27 7.26 7.26 7.25 7.25 7.25 7.24 7.23 7.23 7.22 6.96 6.95 6.95 6.94 6.94 6.93 6.92 6.92 6.91 6.85 6.85 6.85 6.85 6.84 6.83 6.83 6.83 6.82 6.82 6.82 4.80
2.01
1.00
1.98
1.02
f1 (ppm)

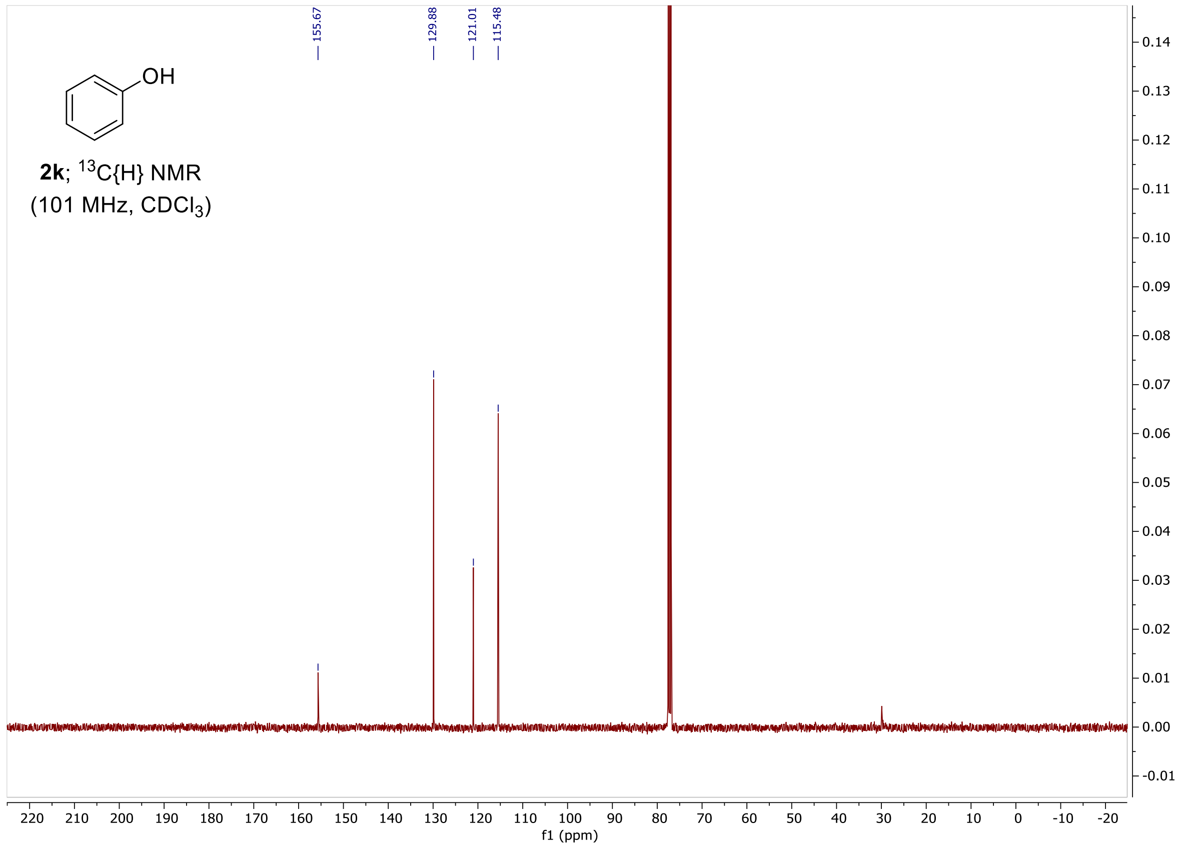
OH
2k; 13C{H} NMR
(101 MHz, CDCl3)
155.67
129.88
121.01
115.48
f1 (ppm)

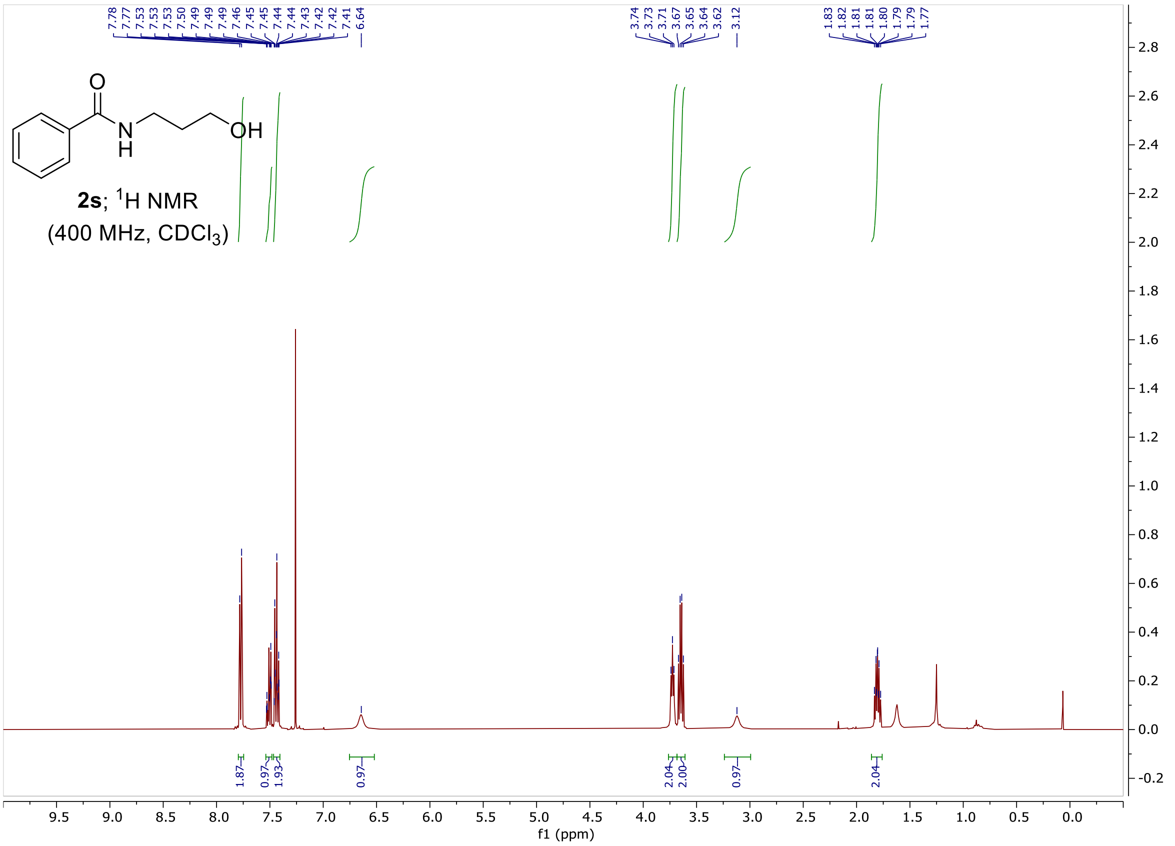

2s; 1H NMR
(400 MHz, CDCl3)
OH
N
H
O
7.78
7.77
7.53
7.53
7.53
7.50
7.49
7.49
7.49
7.46
7.45
7.45
7.44
7.44
7.43
7.42
7.42
7.41
6.64
3.74
3.73
3.71
3.67
3.65
3.64
3.62
3.12
1.83
1.82
1.81
1.81
1.80
1.79
1.79
1.77
1.87
0.97
1.93
0.97
2.04
2.00
0.97
2.04
f1 (ppm)

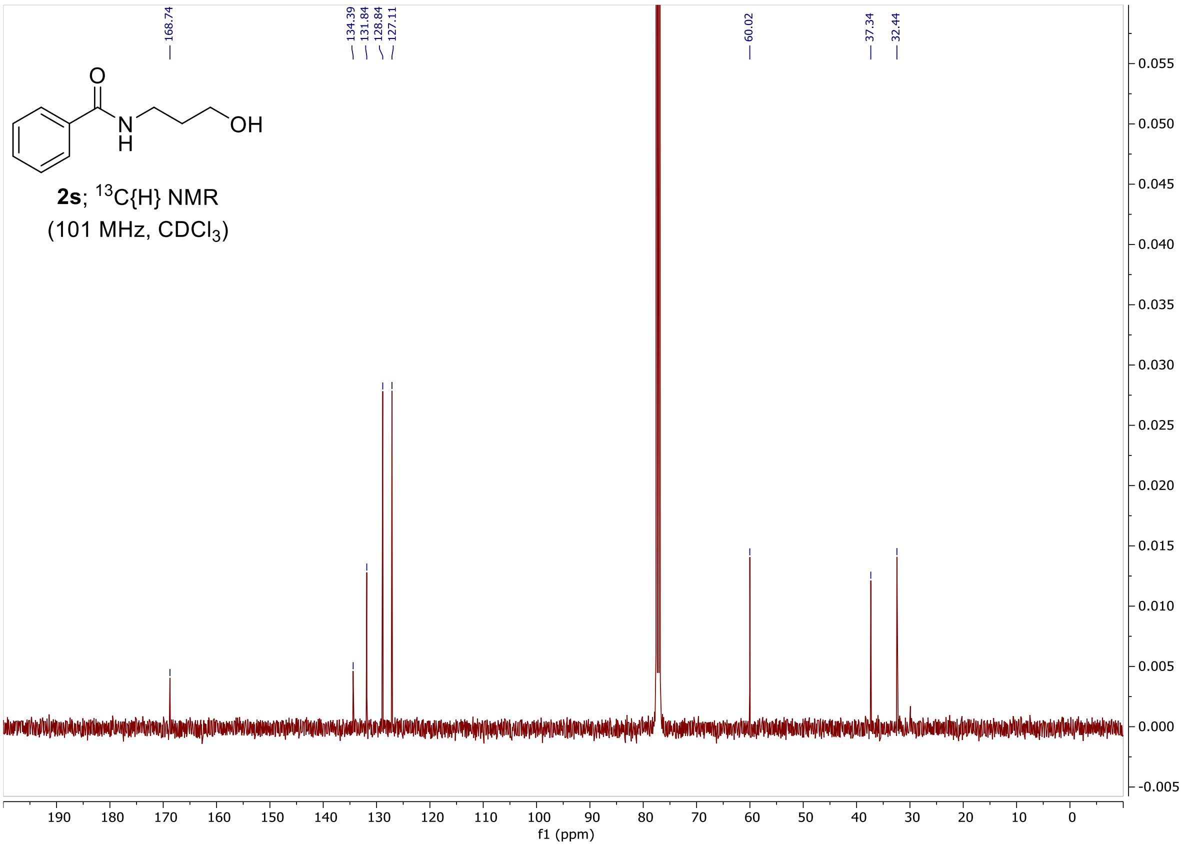

2s; 13C{H} NMR
(101 MHz, CDCl3)
OH
N
H
O
168.74
134.39
131.84
128.84
127.11
60.02
37.34
32.44
f1 (ppm)
